# Nano-ZnO-modified hydroxyapatite whiskers with enhanced osteoinductivity for bone defect repair

**DOI:** 10.1093/rb/rbae051

**Published:** 2024-05-08

**Authors:** Penggong Wei, Ning Wang, Qiyue Zhang, Wanfeng Wang, Hui Sun, Zengqian Liu, Tingting Yan, Qiang Wang, Lihong Qiu

**Affiliations:** School and Hospital of Stomatology, China Medical University, Shenyang 110002, China; Liaoning Provincial Key Laboratory of Oral Diseases, Shenyang 110002, China; Department of Plastic Surgery, The First Hospital of China Medical University, China Medical University, Shenyang 110001, China; School and Hospital of Stomatology, China Medical University, Shenyang 110002, China; Liaoning Provincial Key Laboratory of Oral Diseases, Shenyang 110002, China; School and Hospital of Stomatology, China Medical University, Shenyang 110002, China; Liaoning Provincial Key Laboratory of Oral Diseases, Shenyang 110002, China; School and Hospital of Stomatology, China Medical University, Shenyang 110002, China; Liaoning Provincial Key Laboratory of Oral Diseases, Shenyang 110002, China; Shi-Changxu Innovation Center for Advanced Materials, Institute of Metal Research, Chinese Academy of Sciences, Shenyang 110016, China; Faculty of Materials Science and Engineering, Kunming University of Science and Technology, Kunming 650093, China; School and Hospital of Stomatology, China Medical University, Shenyang 110002, China; Liaoning Provincial Key Laboratory of Oral Diseases, Shenyang 110002, China; School and Hospital of Stomatology, China Medical University, Shenyang 110002, China; Liaoning Provincial Key Laboratory of Oral Diseases, Shenyang 110002, China

**Keywords:** hydroxyapatite whisker, nano-zinc oxide particles surface modification, mechanical damage, osteoblast differentiation, bone defect repair

## Abstract

Hydroxyapatite (HA) whisker (HAw) represents a distinct form of HA characterized by its high aspect ratio, offering significant potential for enhancing the mechanical properties of bone tissue engineering scaffolds. However, the limited osteoinductivity of HAw hampers its widespread application. In this investigation, we observed HAw-punctured osteoblast membranes and infiltrated the cell body, resulting in mechanical damage to cells that adversely impacted osteoblast proliferation and differentiation. To address this challenge, we developed nano-zinc oxide particle-modified HAw (nano-ZnO/HAw). Acting as a reinforcing and toughening agent, nano-ZnO/HAw augmented the compressive strength and ductility of the matrix materials. At the same time, the surface modification with nano-ZnO particles improved osteoblast differentiation by reducing the mechanical damage from HAw to cells and releasing zinc ion, the two aspects collectively promoted the osteoinductivity of HAw. Encouragingly, the osteoinductive potential of 5% nano-ZnO/HAw and 10% nano-ZnO/HAw was validated in relevant rat models, demonstrating the efficacy of this approach in promoting new bone formation *in vivo*. Our findings underscore the role of nano-ZnO particle surface modification in enhancing the osteoinductivity of HAw from a physical standpoint, offering valuable insights into the development of bone substitutes with favorable osteoinductive properties while simultaneously bolstering matrix material strength and toughness.

## Graphical Abstract

**Figure:**
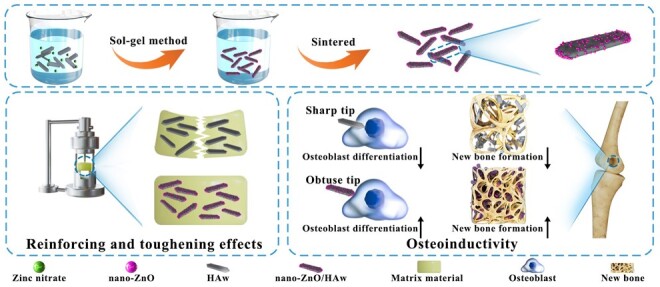

## Introduction

Hydroxyapatite (HA), as a bioactive ceramic, shares a similar chemical composition and structure with the minerals found in natural bone. Its high osteoinductivity, excellent biocompatibility and slow biodegradability have garnered significant attention, positioning synthetic HA as a promising bone substitute material in clinical applications [1]. HA whisker (HAw), characterised by its high aspect ratio along the C-axis, can be synthesized via the hydrothermal homogeneous precipitation method [2]. The unique morphology and properties of HAw render it suitable as a reinforcing and toughening phase, capable of enhancing the mechanical properties of matrix materials by absorbing crack propagation energy through whisker bridging and pulling-out mechanisms. Incorporating HAw into various matrix materials, such as bioactive glass, dental restorative composites or generating HAw *in situ* on the surface of calcium and phosphorus (Ca-P) scaffolds, has been shown to significantly augment their elastic modulus, compressive strength and fracture toughness [3–5]. Furthermore, the mineral composition of natural bone predominantly comprises plate- or whisker-like HA, which aggregates within collagen fibril gaps [6], contributing to the bone’s high fracture resistance [7, 8]. Therefore, synthetic HAw, mirroring the morphology of HA in natural bone, emerges as a promising candidate for bone substitute materials.

Nevertheless, the biocompatibility of HA with high aspect ratios, including HA rods, HAw, HA plates and HA needles, remains a subject of debate. HAw has been reported to inhibit alkaline phosphatase (ALP) activity, calcium deposition and the expression of osteogenic marker genes in osteoblasts or bone marrow mesenchymal stem cells (BMSCs) [5, 9, 10]. HA with larger specific surface areas or higher aspect ratios tends to inhibit cell proliferation [11, 12] and elicit the release of inflammatory factors from cells [13]. HA rods have been shown to increase intracellular reactive oxygen species (ROS) production in liver cells [14], cancer cells [15] and osteoblasts [16], leading to decreased mitochondrial membrane potential, mitochondrial energy metabolism dysfunction and ultimately, cell apoptosis. *In vivo* experiments have demonstrated that intraperitoneal injection of HA rods induces apoptosis in liver and kidney tissues in rats [17]. Moreover, the presence of *in situ*-generated HAw on the surface of Ca-P scaffolds has been associated with reduced new bone formation within the scaffolds following canine intramuscular implantation [5]. Overall, the biocompatibility of HA is shape-dependent, and further investigation is warranted to elucidate the underlying reasons for the reduced biocompatibility of HA with high aspect ratios.

HAw with high specific surface area is a good candidate for carrying various substances [18], including polymer materials [10], metal oxides [19] and metal elements [20, 21]. Polymer-modified HAw shows improved surface properties and higher osteoinductivity compared to unmodified HAw. Furthermore, the chemical combination between polymer materials and polylactic acid matrix materials enhances the mechanical properties of polylactic acid when incorporated with polymer-modified HAw [10]. In osteoporosis rats, bone defects implanted with HAw-modified Ca-P scaffolds exhibit suboptimal bone healing. However, Ca-P scaffolds fabricated with strontium-doped HAw significantly facilitate local bone regeneration and implant osseointegration by releasing strontium. This modification also improves the elastic modulus and hardness of the bone defect [22]. Polymer composite films containing magnesium-doped HAw demonstrate superior hydrophilicity, tensile properties and increased osteoinductivity [20]. The modification with magnesium and strontium on the surface of HAw has been shown to promote the osteoinductivity, Young’s modulus and compressive strength of HAw-based bone tissue engineering scaffold [21, 23]. Collectively, these studies highlight that surface modification with polymer materials or metallic elements can enhance the osteoinductivity of HAw while preserving its reinforcing and toughening effects.

Bioactivity of biomaterials can be enhanced by incorporating biological essential elements such as calcium, phosphorus, magnesium, strontium and zinc (Zn). Zn is a vital trace element in the human body, playing a pivotal role in the synthesis of various enzymes and proteins which are crucial for growth, development and immune function [24, 25]. Zn is considered to be an essential factor for bone development which can promote osteoblasts activity and bone metabolism [26, 27]. Zinc ions (Zn^2+^) serve as activators or co-activators, facilitating the activation of key determinants of osteoblast differentiation, including runt-related transcription factor 2 (RUNX2) and Osterix (OSX) [28]. Implants coated with zinc oxide (ZnO), and bone tissue engineering scaffolds incorporated with Zn or ZnO, have been shown to promote osteoblast differentiation and stimulate new bone formation through the controlled release of Zn^2+^ [29–31]. In addition, Zn is also essential for cellular antioxidant activity. Nuclear factor erythroid 2-related factor (Nrf2), as an antioxidant nuclear transcription factor, can transfer into nucleus after being activated by Zn^2+^ and improve the expression of antioxidant proteins [32]. Biomaterials incorporated with Zn can counteract cellular oxidative stress induced by exogenous materials or endogenous diseases through scavenging intracellular ROS or enhancing cellular antioxidant defenses, thus increasing the osteogenic activity of osteoblasts or BMSCs [30, 33]. Moreover, compared to polymer materials and metallic elements like magnesium and strontium, ZnO exhibits excellent antibacterial properties [34]. ZnO-doped or modified HA has demonstrated promising inhibitory effects against various pathogens, including *Staphylococcus aureus*, *Escherichia coli*, *Candida albicans* and *Streptococcus mutans* [35]. Notably, the oral cavity represents a bacteria-rich environment, with bacterial infections being the leading cause of periapical operation failure. *Porphyromonas gingivalis* (*P.gingivalis*) is a primary pathogen associated with periapical diseases [36, 37]. Therefore, HA with robust antibacterial properties against *P.gingivalis* holds great potential for effectively treating periapical diseases as a commonly utilized periodontal bone substitute material.

In this study, we fabricated nano-ZnO particle-modified HAw (nano-ZnO/HAw) by depositing nano-ZnO particles onto the surface of HAw. We examined the reinforcing and toughening effects of nano-ZnO/HAw on matrix materials. HA spheres (HAs) were used as comparison, the physical interaction between nano-ZnO/HAw and osteoblasts on microscopic characteristics of cells was explored. Furthermore, we systematically explored the effects of HAw and nano-ZnO/HAw on osteoblast differentiation *in vitro* and new bone formation *in vivo*. The primary objective of this study was to elucidate the factors leading to the low osteoinductivity of HAw and to identify effective strategies for enhancing the biocompatibility of HAw.

## Materials and methods

### Fabrication and characterization of nano-ZnO/HAw

HAws were synthesized via the hydrothermal homogeneous precipitation method [2], and served as the substrate material. HAw modified with nano-ZnO particles at mass ratios of 1%, 5% and 10% were prepared using the sol-gel method. Zinc nitrate was used as zinc source. The amount of zinc nitrate and HAw was calculated according to the mass ratio of nano-ZnO particles to HAw as 1%, 5% and 10%. Zinc nitrate was dissolved in an anhydrous ethanol suspension containing HAw. Ammonia and acetic acid were used to keep the pH of the suspension at 7. Then, the anhydrous ethanol in the suspension was evaporated to dryness by a rotary evaporator to obtain the precursors of 1% nano-ZnO/HAw, 5% nano-ZnO/HAw and 10% nano-ZnO/HAw. All the precursors were sintered at 600°C for 10 h. By such means, nano-ZnO/HAw with different proportions of nano-ZnO particles on the surface of HAw was prepared.

The microstructure, size and elemental composition of nano-ZnO/HAw were characterized using scanning electron microscopy (SEM; Zeiss, Germany) and energy dispersive spectroscopy (EDS) analysis.

### Mechanical property evaluation

Tricalcium phosphate and calcium sulfate (Macklin, China) were used as matrix materials. HAs, HAw and 10% nano-ZnO/HAw were, respectively, incorporated into tricalcium phosphate or calcium sulfate at a volume ratio of 10%. Pure tricalcium phosphate or calcium sulfate served as controls. Silica sol (Yanchem, China) was added to tricalcium phosphate, and filled into a mold with a diameter of 10 mm and a height of 8 mm. Deionized water was added to calcium sulfate, and filled into a mold with a diameter of 5 mm and a height of 4 mm. The liquid-solid ratio was 0.5 ml/g. The specimens were loaded with 0.2 mm/min by mechanics experimental machine (Hengyi, China). The maximum bending load and maximum displacement were recorded, the compressive strength and ductility were calculated.

## Antibacterial experiment

### Bacterial culture

*P.gingivalis* standard strains ATCC 33277 (provided by the Department of Oral Biology, School and Hospital of Stomatology, China Medical University, Shenyang, China) were cultured on brain-heart infusion (BHI; Meilun, China) blood agar plates under anaerobic conditions at 37°C. Colonies of *P.gingivalis* were transferred to BHI liquid medium and incubated overnight under the same conditions for subsequent experiments.

### Visual plate counting

*P.gingivalis* (1 × 10^7^ CFU/ml) was co-cultured with suspensions (1 mg/ml) of HAs, HAw, 1% nano-ZnO/HAw, 5% nano-ZnO/HAw and 10% nano-ZnO/HAw in 1.5 ml EP tubes, respectively. Bacteria cultured without HA samples served as the control. After incubating for 24 h, the *P.gingivalis* suspension was vortexed and diluted 10, 100 and 1000 times with PBS. Subsequently, 200 μl of each diluted bacterial suspension was spread and inoculated onto BHI agar plates for 10 days.

### Crystal violet staining

*P.gingivalis* (1 × 10^7^ CFU/ml) was co-cultured with suspensions (1 mg/ml) of HAs, HAw, 1% nano-ZnO/HAw, 5% nano-ZnO/HAw and 10% nano-ZnO/HAw in 24-well plates, respectively. Bacteria cultured without HA samples served as the control. A 316L stainless steel (316L SS) coupon (1 × 1 × 0.3 cm) was placed at the bottom of each well. After incubating for 24 h, the coupons were washed with PBS and fixed in methanol. The sessile bacteria were stained with 0.1% crystal violet staining solution (Meilun, China) for 10 min. The dye attached to the biofilm was eluted with anhydrous ethanol. The eluate was transferred to a 96-well plate, and the absorbance at 600 nm was recorded using a microplate reader (Tecan, Austria).

## *In vitro* cell experiments

### Cell culture

Mouse pre-osteoblastic MC3T3-E1 cells (iCell Bioscience Inc, China) were cultured in alpha minimum essential medium (α-MEM, Meilun, China) supplemented with 10% (v/v) fetal bovine serum (FBS, Clark, USA) and 1% (v/v) penicillin and streptomycin (Gibco, USA) at 37°C, 5% CO_2_. For osteoblast differentiation induction, the medium was supplemented with 50 μg/ml ascorbic acid, 10 mM β-glycerophosphate and 100 nM dexamethasone (Sigma-Aldrich, USA).

### Extract preparation

According to Part 12 of ISO 10993, HAs, HAw, 1% nano-ZnO/HAw, 5% nano-ZnO/HAw and 10% nano-ZnO/HAw with a ratio of 0.1 g/ml were respectively immersed in α-MEM supplemented with 10% FBS in a humidified atmosphere at 37°C with 5% CO_2_. After immersion for 72 h, the suspensions were centrifuged, and the supernatants were collected. Subsequently, the extracts were filtered, diluted 10 times with fresh medium for subsequent experiments.

### Cell morphology characterization

Osteoblasts were seeded in 24-well plates at a density of 1 × 10^5^ per well with a glass coverslip at the bottom of each well. Cells were co-cultured with the suspensions (40 μg/ml) of HAs, HAw and 10% nano-ZnO/HAw, respectively. Cells cultured without HA samples served as the control. After 24 h, the glass coverslips with osteoblasts and HA samples on the surface were fixed with 2.5% glutaraldehyde, then dehydrated in sequential-graded concentrations of ethanol (30%, 50%, 75%, 95% and 100%) for 10 min each. The dehydrated and dried samples were coated with gold-palladium sputtering and observed using SEM (Zeiss, Germany). The elemental compositions of the samples were detected using EDS.

### Lactate dehydrogenase release assay

Osteoblasts were seeded in 96-well plates at a density of 5 × 10^4^ per well. Cells were respectively co-cultured with the suspensions (40 μg/ml) of HAs, HAw, 1% nano-ZnO/HAw, 5% nano-ZnO/HAw and 10% nano-ZnO/HAw in α-MEM with 2.5% FBS. Cells cultured without HA samples served as the control. After incubation for 24 h, the supernatant of each well was transferred to a new 96-well plate. Lactate dehydrogenase (LDH) release assay working solution (Beyotime, China) was added to each well. Following a 30-min incubation, the absorbance at 490 nm was measured using a microplate reader (Tecan, Austria).

### Cell viability assay

Osteoblasts were seeded in 96-well plates at a density of 5 × 10^3^ per well. The extracts or suspensions (40 μg/ml) of HAs, HAw, 1% nano-ZnO/HAw, 5% nano-ZnO/HAw and 10% nano-ZnO/HAw were added to cells, respectively. Cells cultured without HA samples served as the control. After incubation for 24, 48 and 72 h, the supernatants were aspirated. Fresh medium containing 10% CCK-8 solution (Beyotime, China) was added to each well. Following a 2-h incubation at 37°C, the absorbance at 450 nm was measured using a microplate reader (Tecan, Austria).

### ALP staining

Osteoblasts were seeded in 24-well plates at a density of 5 × 10^4^ per well. Cells were co-cultured with the extract or suspension (40 μg/ml) of each sample in differentiation medium. After the corresponding time, cells were fixed with 4% paraformaldehyde for 30 min and then stained with ALP staining solution (Beyotime, China).

### Real-time PCR

Osteoblasts were seeded in 6-well plates at a density of 4 × 10^5^ per well. Cells were co-cultured with suspension (40 μg/ml) of each sample in differentiation medium for 7 days. Total RNA was extracted from cells using TRIzol reagent (Takara, Japan), and cDNA was prepared using the HiScript II Q RT SuperMix for qPCR (Vazyme, China). The expression levels of ALP, RUNX2, type I collagen (COL I), osteocalcin (OCN) and OSX were determined using a 7500 real-time PCR system (Applied Biosystems, USA) with ChamQ Universal SYBR qPCR Master Mix (Vazyme, China). Glyceraldehyde-3-phosphate dehydrogenase (GAPDH) was used as an endogenous control for normalization. The primer sequences are listed in Supplementary Table S1. The relative fold change of gene expression was calculated using the 2^–ΔΔCt^ method.

### Intracellular ROS levels detection

Osteoblasts were seeded in 6-well plates at a density of 4 × 10^5^ per well and co-cultured with suspensions (40 μg/ml) of HAs, HAw, HAw + Zn^2+^ (with the same concentration of Zn^2+^ released from the suspension of 10% nano-ZnO/HAw, as presented in Supplementary Fig. S2) and 10% nano-ZnO/HAw, respectively. Cells were cultured in differentiation medium and incubated for 3 days. The intracellular ROS levels of osteoblasts were detected using a flow cytometer (Becton Dickinson, USA) with an ROS assay kit (Beyotime, China).

### Western blot analysis

Osteoblasts were seeded in 6-well plates at a density of 4 × 10^5^ per well and co-cultured with suspensions (40 μg/ml) of HAs, HAw, HAw + Zn^2+^ and 10% nano-ZnO/HAw in differentiation medium, respectively. After 7 days of incubation, the cells were collected, and the nuclear proteins were extracted using a nuclear and cytoplasmic protein extraction kit (Beyotime, China). The protein samples were separated by SDS-PAGE and transferred onto polyvinylidene fluoride membranes (Merck, Germany). The membranes were blocked with 5% skim milk and then incubated with primary antibodies against Nrf2 (Abclonal, China), H3 and GAPDH (Affinity, USA) overnight at 4°C. Subsequently, the membranes were incubated with secondary antibodies, and the targeted proteins were visualized using an enhanced chemiluminescence detection method.

## *In vivo* animal experiments

### Materials implantation

Approval for all animal experiments was obtained from the Ethics Committee of China Medical University, Shenyang, China (CMU2022039). Eight-week-old male Sprague Dawley rats (Changsheng Biotechnology, China), weighing ∼250 g, were utilized. A cylindrical non-penetrating bone defect with a diameter of 3 mm and a depth of 3 mm was created on the lateral condyle of the rat femur. HAs, HAw, 5% nano-ZnO/HAw and 10% nano-ZnO/HAw were randomly grafted into the bone defect in powder form. Femurs drilled only without implantation served as the blank group. Six parallel samples were employed for each group. Post-operation, the rats received a subcutaneous injection of penicillin for 3 days. Euthanasia was performed on all rats after 4 weeks of implantation using CO_2_ asphyxiation.

### Micro-CT analysis

All the rat femur specimens were scanned by Micro-CT (Scanco medical, Switzerland). The X-ray energy was set at 70 kv and 200 μA, with a resolution of 14.8 μm. The scanned data of each specimen was reconstructed, and the three-dimensional tomography was extracted. A cylindrical volume of interest (VOI) with a diameter of 3 mm and a height of 3 mm was selected from the surface of cortical bone to cancellous bone, which was corresponding to the dimension of the bone defect. Components within the VOI consistent with the density of bone tissue were selected (threshold was set as 65–150) and reconstructed using an image analysis software.

### Histological staining

The harvested specimens underwent decalcification in 10% ethylene diamine tetraacetic acid for 8 weeks. Subsequently, the specimens were dehydrated in graded ethanol, embedded in paraffin and sectioned. The sections were stained using haematoxylin and eosin (H&E) staining solution (Beyotime, China). Histological images were scanned using a digital scanner (Shengqiang, China).

### Statistical analysis

All quantitative data were presented as mean ± standard deviation (SD) as indicated and analyzed using one-way analysis of variance (ANOVA) with a Tukey’s test for multiple comparisons or t test for two-group comparisons. Asterisks denoted statistically significant differences (* *P *<* *0.05; ** *P *<* *0.01; ns, no significance).

## Results

### Characterization of nano-ZnO/HAw

**Figure 1.**
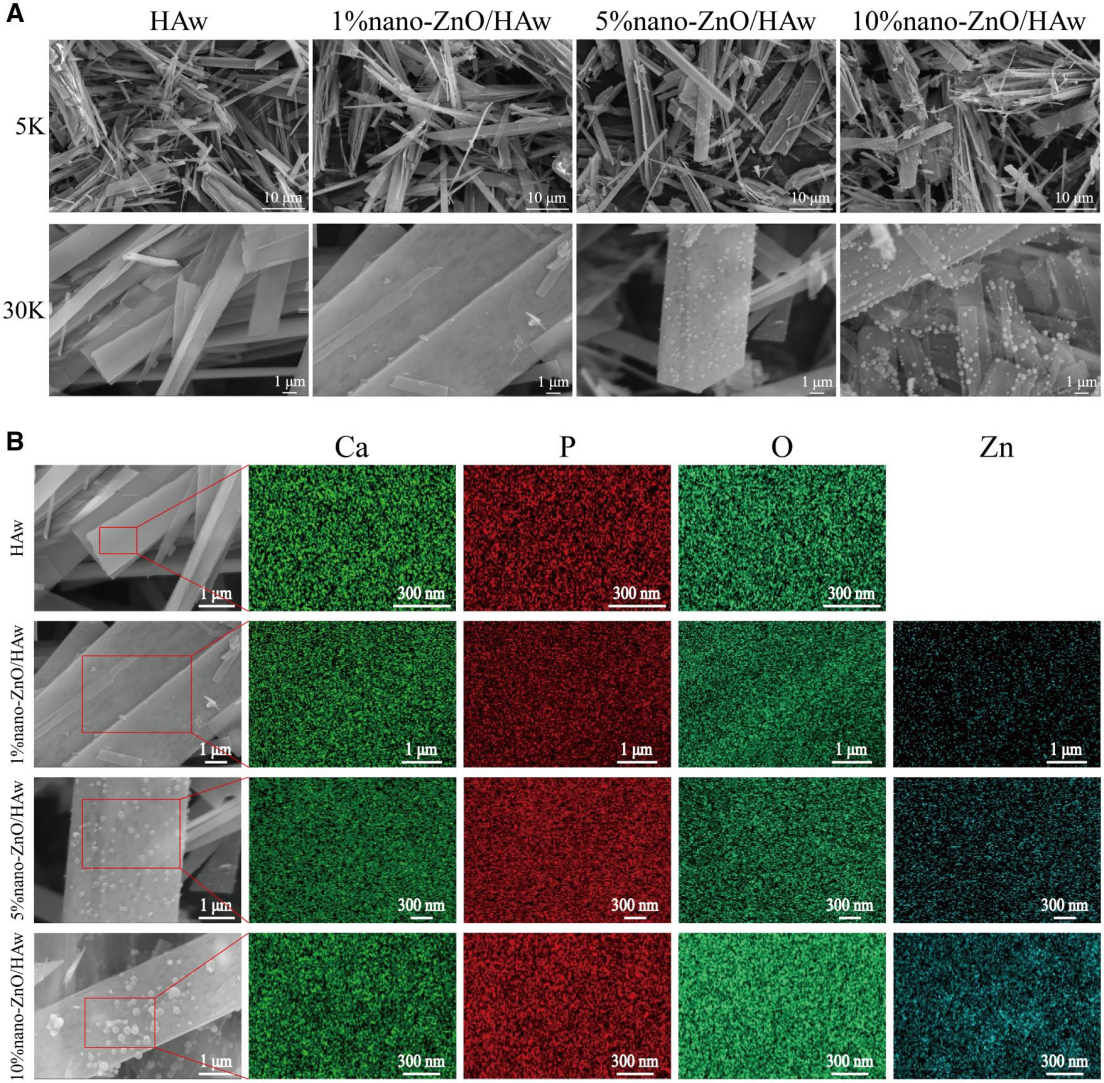
Characterization of nano-ZnO/HAw. (**A**) Microstructure and (**B**) elemental composition of HAw, 1% nano-ZnO/HAw, 5% nano-ZnO/HAw and 10% nano-ZnO/HAw.

HAw modified with 1%, 5% and 10% nano-ZnO particles was successfully fabricated. The phase compositions of HAw and nano-ZnO/HAw were characterised by X-ray diffraction. As depicted in Supplementary Fig. S1, the phase composition of pure HAw was HA, while nano-ZnO/HAw comprised HA and ZnO phases. The crystalline structure of ZnO was evident in nano-ZnO/HAw, with the peak strength of ZnO gradually increasing in proportion to the nano-ZnO content. The precise nano-ZnO contents in HAw, 1% nano-ZnO/HAw, 5% nano-ZnO/HAw and 10% nano-ZnO/HAw were determined as 0%, 1.59 ± 0.01%, 7.35 ± 0.03% and 14.50 ± 0.09%, respectively (Supplementary Table S2). The release behavior of Zn^2+^ from nano-ZnO/HAw was investigated using inductively coupled plasma mass spectrometry. No Zn^2+^ was detected from HAw alone, whereas Zn^2+^ release was observed from nano-ZnO/HAw, with higher level from 10% nano-ZnO/HAw compared to 1% nano-ZnO/HAw and 5% nano-ZnO/HAw (Supplementary Fig. S2). The morphologies (Fig. 1A) and sizes (Supplementary Table S3) of nano-ZnO/HAw were characterized by SEM. Pure HAw displayed a smooth surface with sharp tips, with an average length of ∼13.37 ± 2.38 μm and a width of 1.68 ± 0.46 μm. Upon modification with nano-ZnO particles, the surface of HAw became coated with these particles, distributed uniformly. With increasing proportions of nano-ZnO particles, the wrapping of HAw became more complete. The sizes of 1% nano-ZnO/HAw and 5% nano-ZnO/HAw were similar to those of HAw. However, 10% nano-ZnO/HAw had a relatively smaller length compared to pure HAw (10.85 ± 1.89 μm vs 13.37 ± 2.38 μm), while the width was comparable to that of HAw. The aspect ratios of HAw, 1% nano-ZnO/HAw, 5% nano-ZnO/HAw and 10% nano-ZnO/HAw were 8.52 ± 2.68, 8.86 ± 2.91, 8.19 ± 2.81 and 7.43 ± 2.92, respectively. The elemental compositions of nano-ZnO/HAw were identified by EDS, revealing Ca, P, O and Zn elements (Fig. 1B). The Zn proportions on the surface of HAw, 1% nano-ZnO/HAw, 5% nano-ZnO/HAw and 10% nano-ZnO/HAw were 0%, 0.57%, 5.12% and 12.40%, respectively (Supplementary Table S4).

### Mechanical property

**Figure 2.**
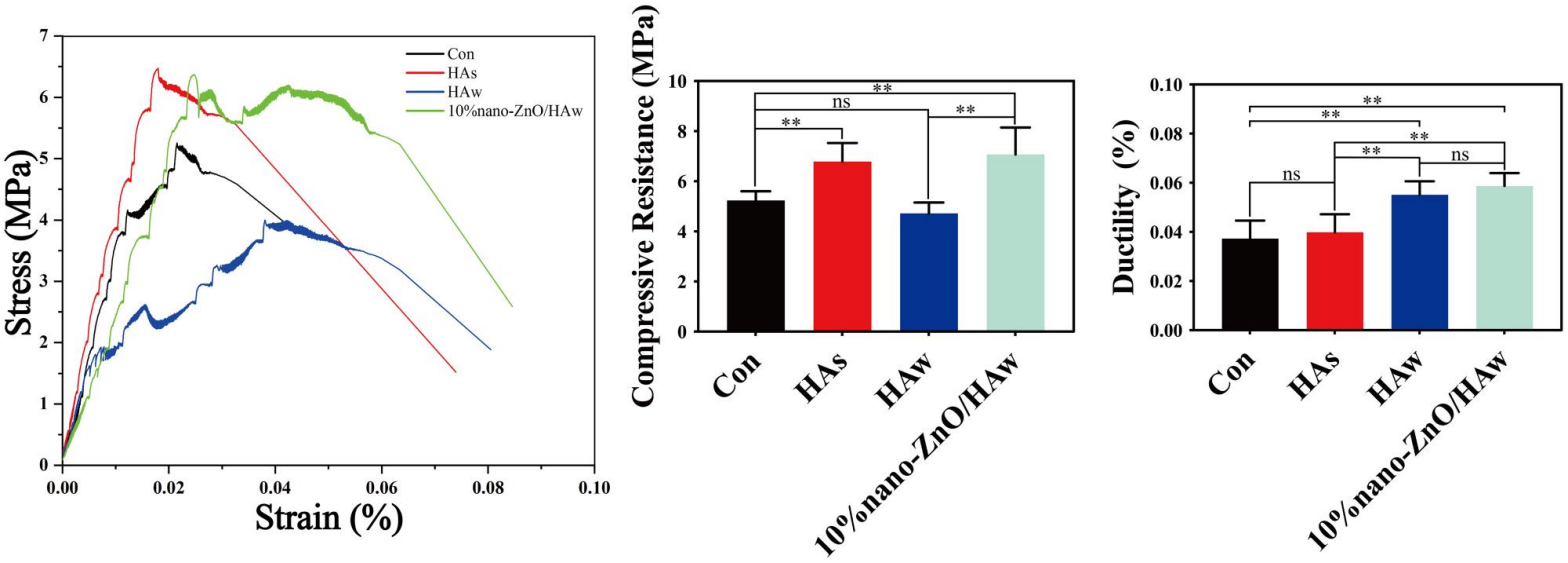
Compressive strength and ductility of tricalcium phosphate incorporated with HAs, HAw and 10% nano-ZnO/HAw (**P <* *0.01; ns, no significance).

Tricalcium phosphate and calcium sulfate are commonly employed for bone defect repair in clinical settings [38], and served as matrix materials to assess the reinforcing and toughening effects of nano-ZnO/HAw in this study. HAs, HAw and 10% nano-ZnO/HAw were individually incorporated into tricalcium phosphate or calcium sulfate. The compressive strength of tricalcium phosphate incorporated with HAw was comparable to that of pure tricalcium phosphate. However, compared to the control or the HAs group, the ductility of tricalcium phosphate significantly increased in the HAw group. Notably, both the compressive strength and ductility of tricalcium phosphate incorporated with 10% nano-ZnO/HAw surpassed those of pure tricalcium phosphate (Fig. 2). Similarly, the ductility of calcium sulfate incorporated with HAw and 10% nano-ZnO/HAw displayed an increasing trend compared to the control or the HAs group. The compressive strength of calcium sulfate incorporated with HAw or 10% nano-ZnO/HAw exceeded that of pure calcium sulfate. Calcium sulfate incorporated with 10% nano-ZnO/HAw exhibited the highest compressive strength among all groups, surpassing even that of the HAw group (Supplementary Fig. S3). In summary, the incorporation of nano-ZnO/HAw effectively enhanced the mechanical properties of the matrix materials.

### Antibacterial property of nano-ZnO/HAw

**Figure 3.**
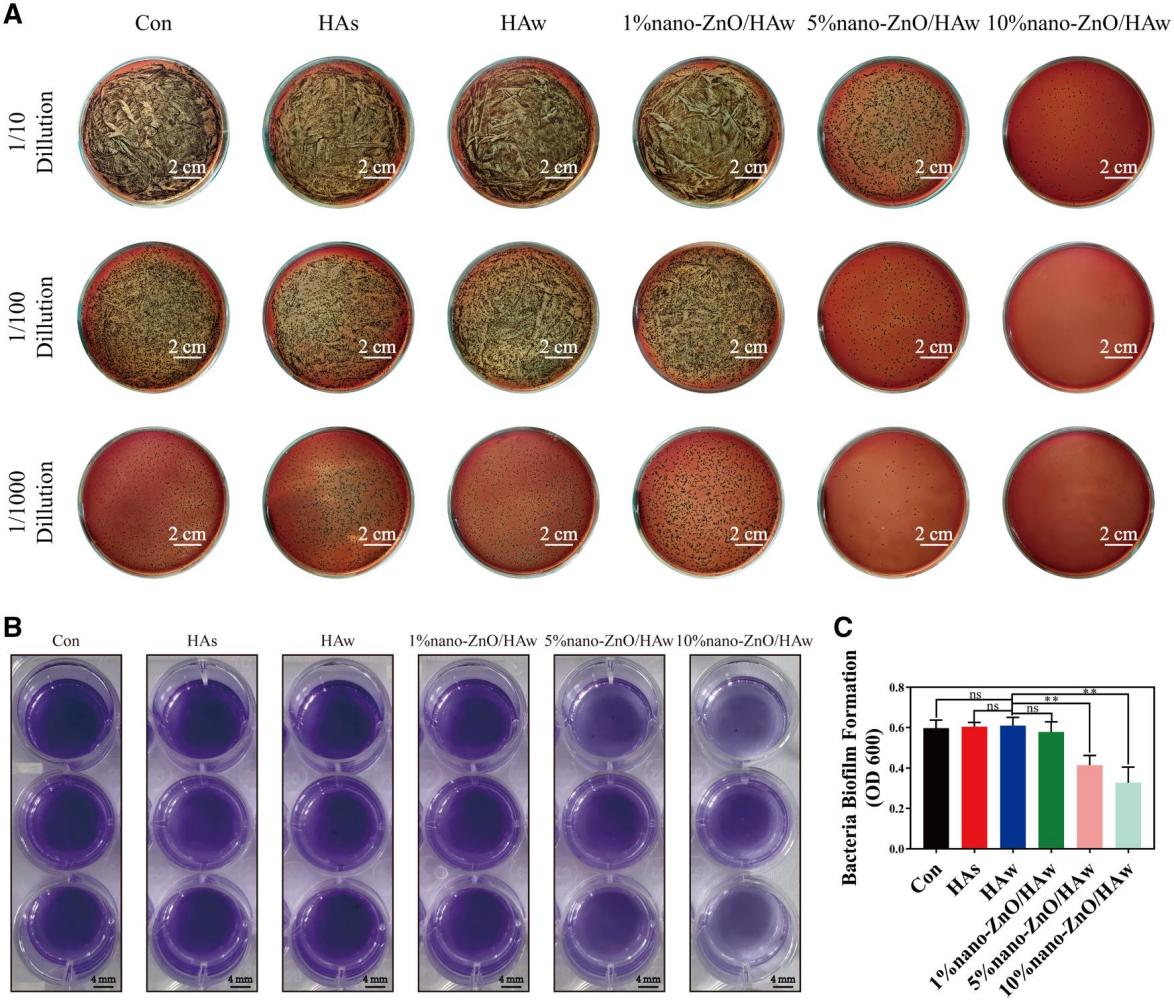
Bacterial viability and biofilm formation of *P.gingivalis* co-cultured with nano-ZnO/HAw. (**A**) Colony formation and (**B**, **C**) biofilm formation of *P.gingivalis* co-cultured with the suspension of HAs, HAw and nano-ZnO/HAw (**P <* *0.01; ns, no significance).

Visual plate counting (Fig. 3A) and crystal violet staining (Fig. 3B and C) were conducted to evaluate the impact of nano-ZnO/HAw on *P.gingivalis* viability and biofilm formation. No significant differences in *P.gingivalis* colony formation and biofilm formation were observed among the control, HAs and HAw groups. The colony formation and biofilm formation of *P.gingivalis* in the 1% nano-ZnO/HAw group were comparable to those in the HAw group. Conversely, the colony formation and biofilm formation of *P.gingivalis* significantly decreased in the 5% nano-ZnO/HAw and 10% nano-ZnO/HAw groups compared to the HAw group.

### Microscopic characteristics and membrane integrity of osteoblasts

**Figure 4.**
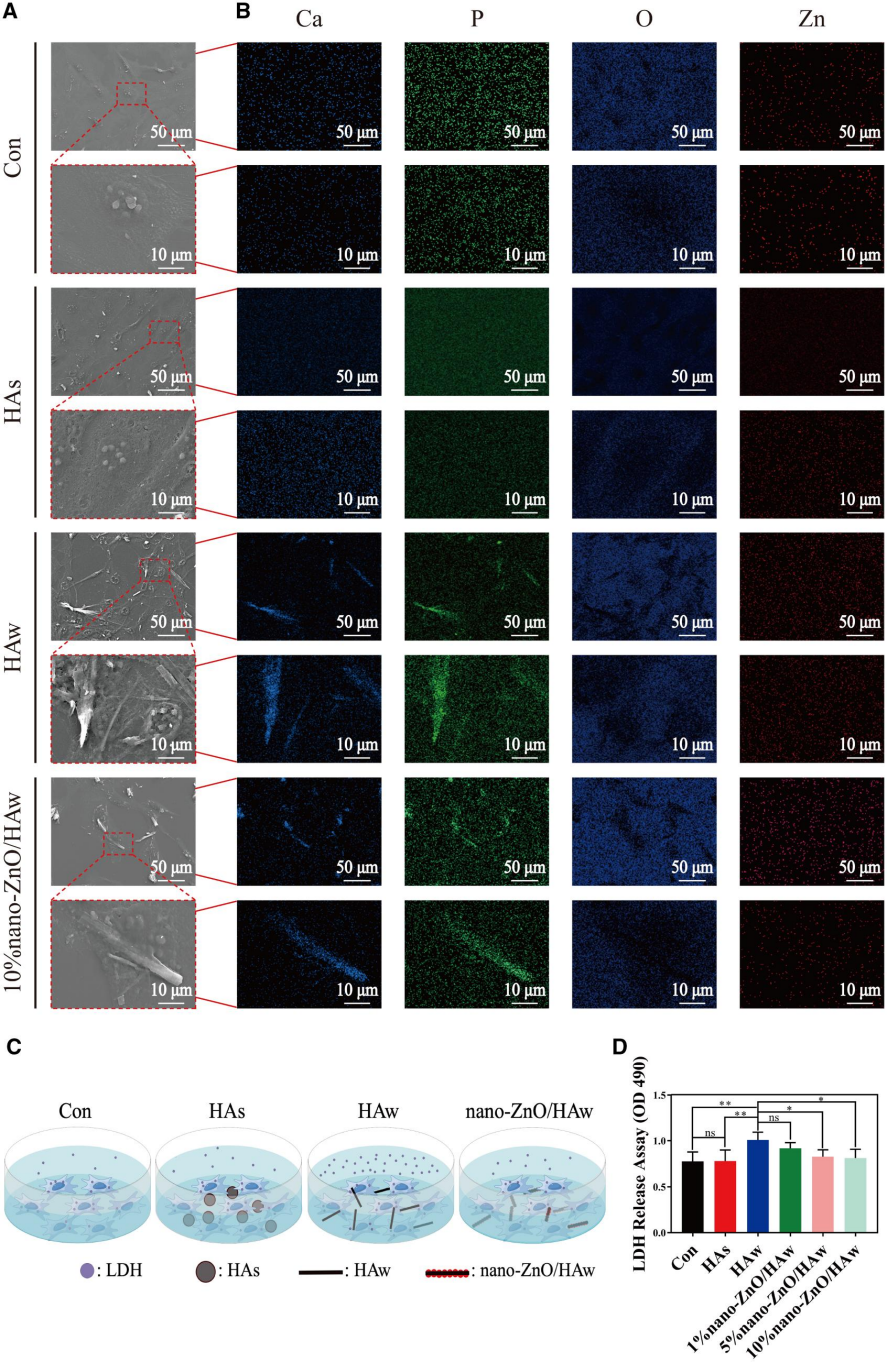
Microscopic characteristics and membrane integrity of osteoblasts co-cultured with HAw and nano-ZnO/HAw. (**A**) Microscopic characteristics and (**B**) elemental compositions of osteoblasts co-cultured with HAs, HAw and 10% nano-ZnO/HAw; (**C**) strategy diagram for osteoblasts co-cultured with the suspension of HAs, HAw and nano-ZnO/HAw; (**D**) LDH leakage of osteoblasts co-cultured with different materials (*P <* *0.05; **P <* *0.01; ns, no significance).

The effect of the physical interaction between nano-ZnO/HAw and osteoblasts on cells microscopic characteristics was analyzed using SEM and EDS (Fig. 4A and B). Osteoblasts exhibited proper stretching in the control and HAs groups. Interestingly, it could be captured that HAw and 10% nano-ZnO/HAw punctured osteoblasts membrane and inserted into cell body when cells were co-cultured with the suspensions above. Nevertheless, the punctured cells could not be observed in HAs group (Fig. 4A). EDS analysis revealed high-intensity Ca-P signals with a high aspect ratio in the regions where HAw and 10% nano-ZnO/HAw were inserted into osteoblasts, confirming the presence of HAw (Fig. 4B). LDH is a relatively stable enzyme in cells which can be released into culture medium with the rupture of cell membrane. The released amount of LDH is regarded as an important indicator of cell membrane integrity [39]. As shown in Fig. 4C, osteoblasts were co-cultured with the suspensions of HAs and nano-ZnO/HAw, respectively. The integrity of osteoblasts membrane was quantitatively analyzed by LDH release assay. There was no significant difference in LDH release between the control and HAs groups. However, the release of LDH was higher in the HAw group compared to the control or HAs groups. Notably, the release of LDH decreased significantly in the 5% nano-ZnO/HAw and 10% nano-ZnO/HAw groups compared to the HAw group (Fig. 4D). Trypan blue staining assay was performed to further compare the integrity of osteoblasts membrane. Once the trypan blue dye entered cells, it indicated that the cell membrane integrity was disrupted [40]. The results showed that the number of blue-stained cells in the HAs group was similar to that in the control group. In contrast, there were more blue-stained cells in the HAw group compared to the control group. However, fewer blue-stained cells were observed in the 5% nano-ZnO/HAw or 10% nano-ZnO/HAw groups compared to the HAw group (Supplementary Fig. S4).

### Biocompatibility of HAw and nano-ZnO/HAw *in vitro*

**Figure 5.**
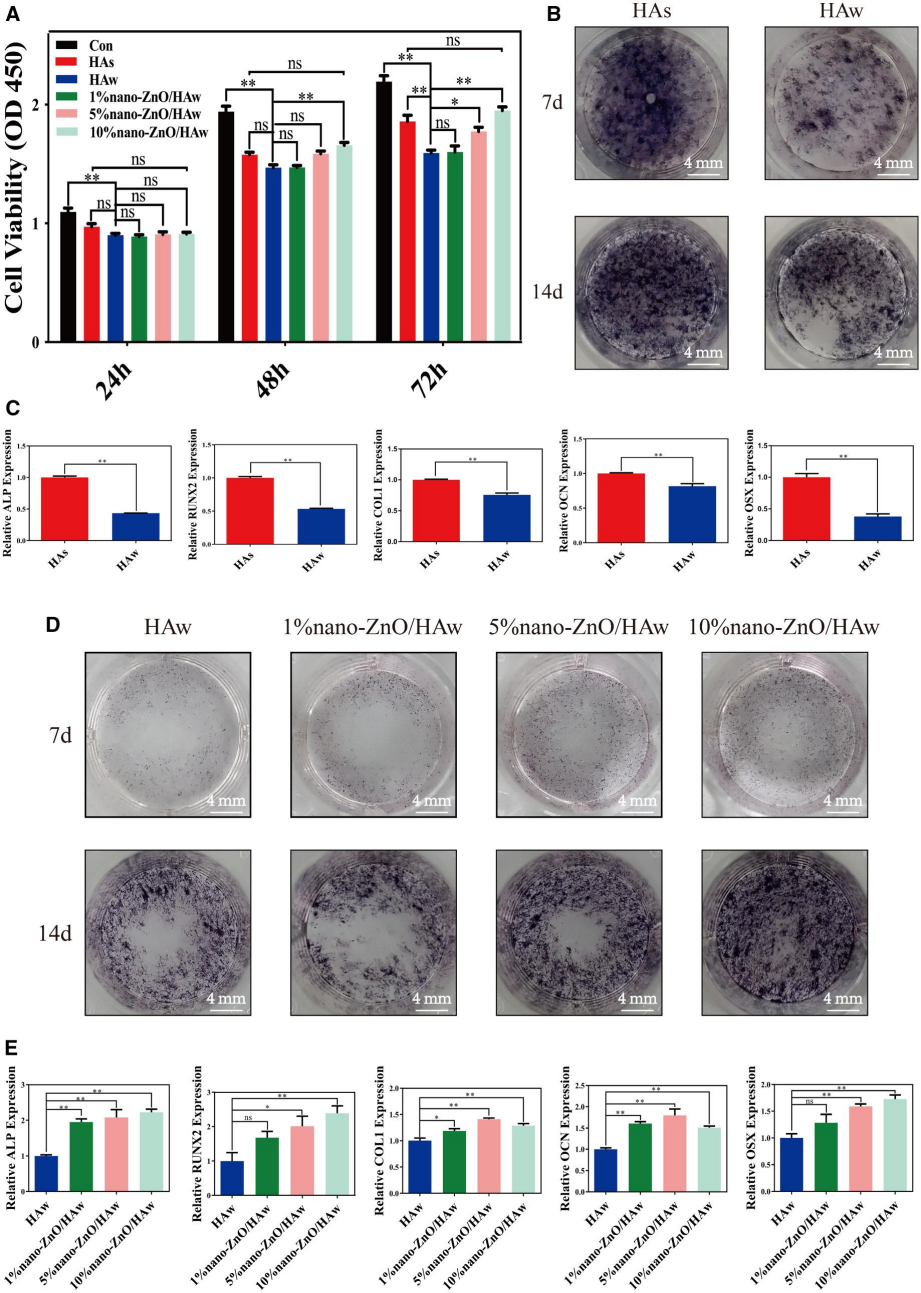
Osteoblast proliferation and differentiation of cells co-cultured with HAw and nano-ZnO/HAw. (**A**) Cell viability of osteoblasts co-cultured with the suspension of HAs, HAw and nano-ZnO/HAw for 24, 48 and 72 h; (**B**) ALP activity and (**C**) osteogenic marker genes expression of osteoblasts co-cultured with the suspension of HAs and HAw for 7 and/or 14 days; (**D**) ALP activity and (**E**) osteogenic marker genes expression of osteoblasts co-cultured with the suspension of HAw, 1% nano-ZnO/HAw, 5% nano-ZnO/HAw and 10% nano-ZnO/HAw for 7 and/or 14 days (*P <* *0.05; **P <* *0.01; ns, no significance).

Biocompatibility assessment is crucial for determining the suitability of biomaterials. Previous studies have highlighted the shape-dependent biocompatibility of HA, with higher aspect ratios or larger specific surface areas correlating to lower biocompatibility [5, 9]. As a bone substitute, HAs was commonly used in clinics [1], and served as a reference to evaluate the cytocompatibility and osteoinductivity of HAw and nano-ZnO/HAw in this study. The CCK8 assay revealed that HAw significantly inhibited osteoblast proliferation compared to the control group at 24, 48 and 72 h, as well as compared to the HAs group at 72 h (Fig. 5A). Moreover, osteoblasts co-cultured with HAw exhibited a higher apoptosis rate compared to those co-cultured with HAs (Supplementary Fig. S5A and B). Additionally, ALP staining and real-time PCR analysis demonstrated that HAw suppressed the ALP activity (Fig. 5B) and the expression of osteogenic marker genes, including ALP, RUNX2, COL I, OCN and OSX (Fig. 5C) in osteoblasts at 7 and/or 14 days, relative to HAs.

Surface modification represents an effective strategy to enhance the biocompatibility of HAw. Previous studies have shown that modifications with polymer materials or metallic elements such as magnesium and strontium improved the cytocompatibility and osteoinductivity of HAw [10, 21, 22]. In this study, the biocompatibility of HAw modified with varying proportions of nano-ZnO particles was assessed. The surface modification with 5% and 10% nano-ZnO particles effectively mitigated the inhibitory effect of HAw on osteoblast proliferation at 48 or 72 h. And the osteoblast proliferation in the 10% nano-ZnO/HAw group was comparable to that of HAs group at 24, 48 and 72 h (Fig. 5A). Furthermore, apoptosis of osteoblasts induced by HAw was reduced with the modification of 1%, 5% and 10% nano-ZnO particles (Supplementary Fig. S5A and B). Compared to pure HAw, nano-ZnO/HAw significantly enhanced the ALP activity (Fig. 5D) and the expression of osteogenic marker genes, including ALP, RUNX2, COL I, OCN and OSX (Fig. 5E) in osteoblasts at 7 and/or 14 days. Notably, the surface modification with 5% and 10% nano-ZnO particles exhibited a more pronounced effect on enhancing the cytocompatibility and osteoinductivity of HAw compared to 1% nano-ZnO particles.

### Effects of indirect contact between nano-ZnO/HAw and osteoblasts on cell proliferation and differentiation

**Figure 6.**
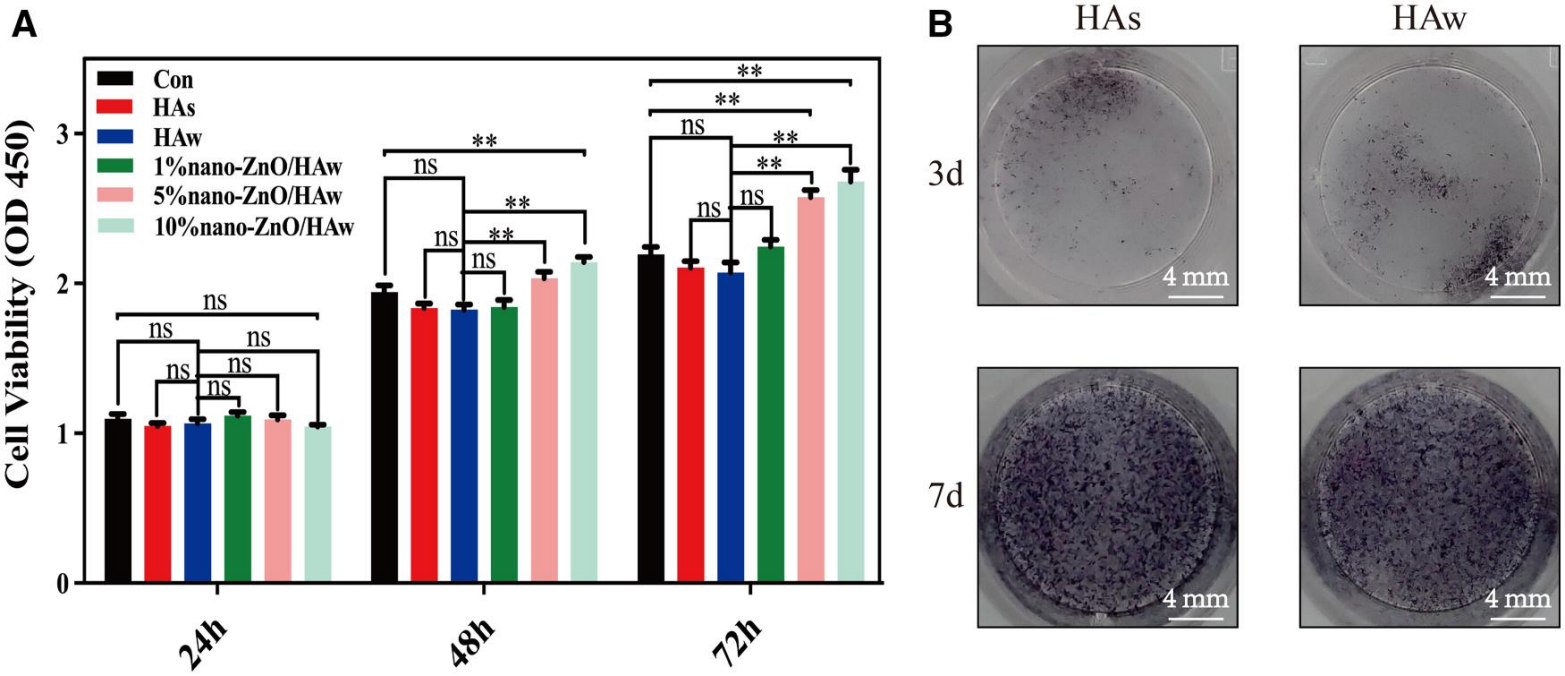
Osteoblast proliferation and ALP activity of cells co-cultured with the extract of HAw. (**A**) Cell viability of osteoblasts co-cultured with the extract of HAs, HAw and nano-ZnO/HAw for 24, 48 and 72 h; (**B**) ALP activity of osteoblasts co-cultured with the extract of HAs and HAw for 3 and 7 days (**P <* *0.01; ns, no significance).

To analyze the impact of the physical morphologies of HAw and nano-ZnO/HAw on their biocompatibility, extracts of HAw and nano-ZnO/HAw were prepared and co-cultured with osteoblasts. This study compared the effects of direct contact (Fig. 5A–E) and indirect contact (Fig. 6A and B) between nano-ZnO/HAw and osteoblasts on cell proliferation and differentiation. As depicted in Fig. 6A, there were no significant differences in osteoblast proliferation among the control, HAs and HAw groups at 24, 48 and 72 h. The proliferation of osteoblasts co-cultured with the extract of 1% nano-ZnO/HAw was comparable to that of pure HAw. Intriguingly, compared with the extract of pure HAw and the control group, the extract of 5% nano-ZnO/HAw and 10% nano-ZnO/HAw significantly promoted osteoblast proliferation at 48 or 72 h. The ALP activity of osteoblasts co-cultured with the extract of HAw or nano-ZnO/HAw was also assessed. No significant difference in osteoblast ALP activity was observed between the HAs and HAw groups at 3 and 7 days (Fig. 6B). However, compared with the extract of pure HAw, the extract of nano-ZnO/HAw noticeably promoted osteoblast ALP activity. Particularly, the extract of 10% nano-ZnO/HAw exhibited a higher promoting effect on osteoblast ALP activity than the extracts of 1% nano-ZnO/HAw and 5% nano-ZnO/HAw at 3 and 7 days (Supplementary Fig. S6).

### Intracellular ROS production and endonuclear Nrf2 expression of osteoblasts

**Figure 7.**
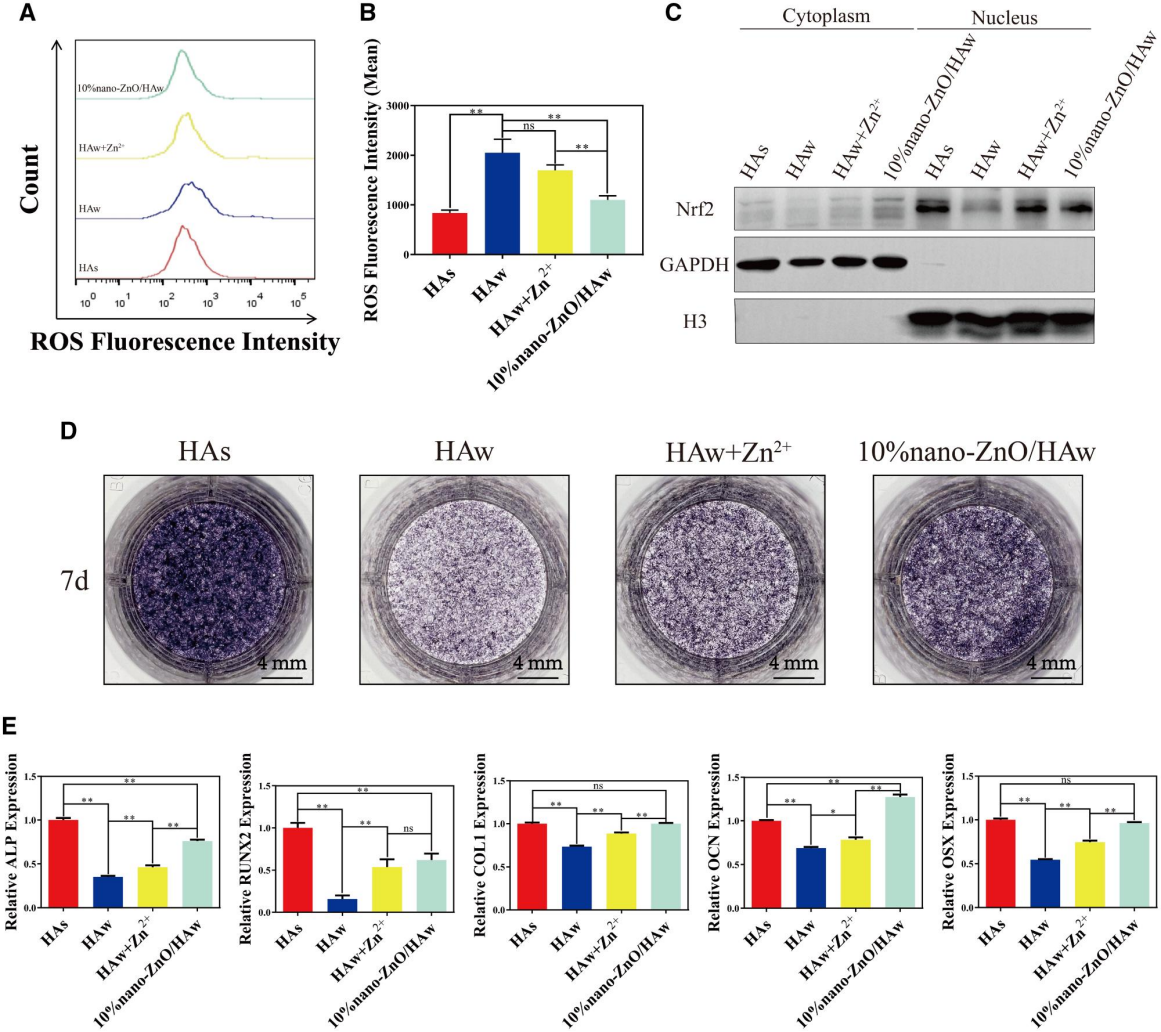
Effects of the released Zn^2+^ on endonuclear Nrf2 expression and differentiation of osteoblasts. (**A**, **B**) Intracellular ROS level of osteoblasts co-cultured with the suspension of HAs, HAw, HAw + Zn^2+^ and 10% nano-ZnO/HAw for 3 days; (**C**) endonuclear Nrf2 expression, (**D**) ALP activity and (**E**) osteogenic marker genes expression of osteoblasts co-cultured with the suspension of HAs, HAw, HAw + Zn^2+^ and 10% nano-ZnO/HAw for 7 days (* *P *<* *0.05; ** *P *<* *0.01; ns, no significance).

Previous studies have indicated that high aspect ratio surface topographies contacting with bacteria or penetrating bacteria result in elevated ROS levels in bacteria [41, 42]. In this study, evidence of HAw penetration into osteoblasts was observed (Fig. 4A and B), prompting further investigation into the effects of HAw and 10% nano-ZnO/HAw on intracellular ROS production in osteoblasts. As depicted in Fig. 7A and B, compared to HAs, HAw significantly increased intracellular ROS production in osteoblasts. Conversely, osteoblasts co-cultured with the suspension of 10% nano-ZnO/HAw exhibited lower intracellular ROS levels than those co-cultured with HAw. Nrf2 is essential to cellular antioxidant function. Studies have reported that Nrf2 played a regulatory role in osteogenic differentiation of BMSCs and bone formation [43, 44]. The impact of HAw and 10% nano-ZnO/HAw on endonuclear Nrf2 expression in osteoblasts was assessed using Western blot analysis. As illustrated in Fig. 7C, compared to HAs, HAw reduced endonuclear Nrf2 expression in osteoblasts. However, osteoblasts in the 10% nano-ZnO/HAw group exhibited higher endonuclear Nrf2 expression compared to the HAw group.

Nrf2 can be activated by Zn^2+^ and translocate to nucleus, thereby activating the transcription of multiple antioxidant proteins in response to various kinds of stress [32]. The influence of Zn^2+^ released from 10% nano-ZnO/HAw on intracellular ROS production and endonuclear Nrf2 expression in osteoblasts was investigated. Osteoblasts were co-treated with HAw and the same concentration of Zn^2+^ released from the suspension of 10% nano-ZnO/HAw. Compared to osteoblasts treated with HAw alone, those co-treated with HAw and Zn^2+^ exhibited a decrease in intracellular ROS production (Fig. 7A and B) and an increase in endonuclear Nrf2 expression (Fig. 7C). Furthermore, the effect of released Zn^2+^ on the osteoinductivity of HAw was assessed. Compared to the HAw group, the supplementation of Zn^2+^ into the HAw suspension significantly increased the ALP activity (Fig. 7D) and the expression of osteogenic marker genes, including ALP, RUNX2, COL I, OCN and OSX (Fig. 7E) in osteoblasts. Additionally, the inducibility of HAs and 10% nano-ZnO/HAw on osteoblast differentiation was compared. The ALP activity and the expression of ALP and RUNX2 genes were lower in the 10% nano-ZnO/HAw group than those in the HAs group. However, compared to the HAs group, the expression of COL I and OSX genes was similar in the 10% nano-ZnO/HAw group, while the expression of OCN gene was increased (Fig. 7D and E).

### Osteoinductivity of HAw and nano-ZnO/HAw *in vivo*

**Figure 8.**
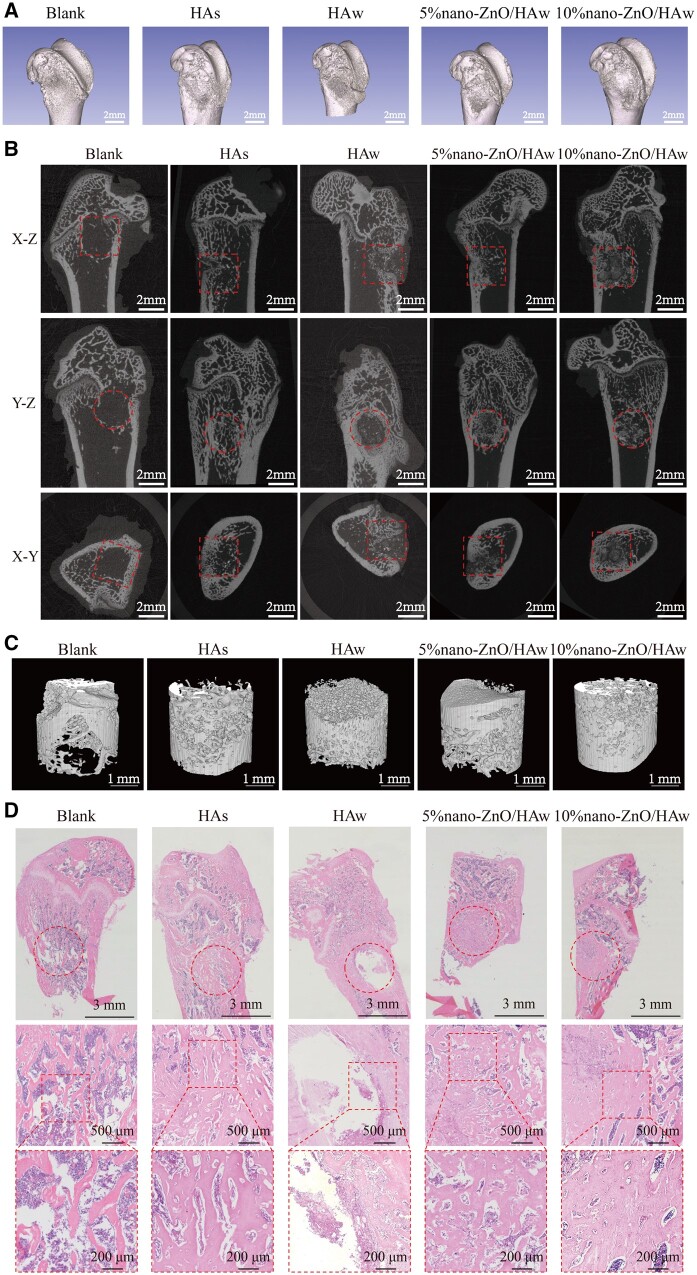
Osteoinductivity of nano-ZnO/HAw *in vivo*. HAw and nano-ZnO/HAw were grafted into bone defect for 4 weeks. (**A**) Three-dimensional reconstruction of femur in relevant bone defect models; (**B**) three-dimensional tomography of rat femur; (**C**) total volume of bone tissue-like components in cylindrical bone defect; (**D**) H&E staining of bone defect area.

Previous studies have indicated that HAw exhibits poor osteoinductivity *in vivo*. For instance, HAw generated *in situ* on the surface of Ca-P scaffolds led to reduced new bone formation inside the scaffolds after canine intramuscular implantation [5]. However, the impact of HAw used as a bone substitute on new bone formation in bone defects has not been extensively reported. In this study, HAw and nano-ZnO/HAw were implanted into rat femoral defects to assess the osteoinductivity of nano-ZnO/HAw. After 4 weeks of implantation, new bone formation within the bone defects was evaluated using Micro-CT and H&E staining. The three-dimensional reconstruction results revealed that the cortical bone defects in the blank group and HAs group were essentially healed. In contrast, the cortical bone defects in the HAw group exhibited significant unhealed areas (Fig. 8A). Additionally, the cancellous bone defects in the blank group showed low-density images with minimal bone ingrowth, while those grafted with HAs exhibited higher-density images compared to the HAw group (Fig. 8B). The total volume of bone tissue-like components in the HAs group was higher than that in the HAw group (Fig. 8C). The percent bone volumes (BV/TV), trabecular number (Tb.N), trabecular thickness (Tb.Th) and trabecular separation (Tb.Sp) were quantitatively calculated to analyze bone regeneration within the bone defects. The BV/TV of the HAs group was higher than that of the HAw group. However, there were no significant differences in Tb.N, Tb.Th and Tb.Sp among the blank, HAs and HAw groups (Supplementary Fig. S7). H&E staining corroborated these findings, with the HAs group exhibiting greater new bone formation compared to the blank group. Conversely, the HAw group showed clear empty holes with minimal new bone tissue ingrowth (Fig. 8D). These results confirmed that HAw inhibited new bone formation in bone defect, indicating the poor osteoinductivity of HAw *in vivo*, which was consistent with the results that HAw decreased osteoblast differentiation *in vitro* (Fig. 5B and C).

In comparison to the HAw group, the cortical bone defects healed better (Fig. 8A), and the bone defect areas showed higher image densities (Fig. 8B) in the 5% nano-ZnO/HAw and 10% nano-ZnO/HAw groups. Moreover, the total volume of bone tissue-like components in the bone defects of these groups was higher than that in the HAw group (Fig. 8C). Quantitative analysis revealed higher BV/TV and/or Tb.Th in the 5% nano-ZnO/HAw and 10% nano-ZnO/HAw groups compared to the HAw group (Supplementary Fig. S7). H&E staining further supported these findings, showing increased new bone formation in the 5% nano-ZnO/HAw and 10% nano-ZnO/HAw groups compared to the HAw group (Fig. 8D). These results indicated that the surface modification with 5% and 10% nano-ZnO particles improved the inducibility of HAw on new bone formation in bone defect. This was consistent with the results that nano-ZnO particles surface modification improved the inducibility of HAw on osteoblasts differentiation *in vitro* (Fig. 5D and E). Meanwhile, the new bone formation of HAs, 5% nano-ZnO/HAw and 10% nano-ZnO/HAw groups was at a same level, which was higher than that of blank group (Fig. 8A–D), demonstrating that 5% and 10% nano-ZnO particles surface modification increased the osteoinductivity of HAw *in vivo*, 5% nano-ZnO/HAw and 10% nano-ZnO/HAw could promote new bone formation.

## Discussion

HAw is commonly utilized as a reinforcing and toughening phase to enhance the mechanical properties of bone tissue engineering scaffolds [3, 45]. In this study, we fabricated HAw and nano-ZnO/HAw. Pure HAw displayed a smooth surface with a sharp tip, while the addition of nano-ZnO particles resulted in the formation of a ‘nano-ZnO particle film’ on the surface of HAw (Fig. 1A). We verified the reinforcing and toughening effects of nano-ZnO/HAw on the mechanical properties of matrix materials. The ductility of tricalcium phosphate or calcium sulfate incorporated with HAw was higher than that of the control group and the HAs group (Fig. 2A and Supplementary Fig. S3), indicating that HAw contributed to enhancing the ductility of the matrix materials. Nano-ZnO particles played a crucial role as secondary enhancers. Previous studies have shown that the compressive strength and diametral tensile strength of dental cement increased significantly with the addition of ZnO particles [46]. Similarly, in our study, tricalcium phosphate incorporated with 10% nano-ZnO/HAw exhibited higher compressive strength and ductility than pure tricalcium phosphate (Fig. 2A). Calcium sulfate incorporated with 10% nano-ZnO/HAw exhibited the highest compressive strength among all the groups, surpassing even that of the HAw group (Supplementary Fig. S3). This suggests that HAw and nano-ZnO particles served as secondary enhancers jointly promoted the mechanical properties of tricalcium phosphate and calcium sulfate, proving that nano-ZnO/HAw had better potential to improve the mechanical properties of matrix materials.

Periapical diseases are primary causes of tooth loss and jawbone absorption, often necessitating periapical operations for removal. However, the persistence of residual bacteria, compounded by the bacteriogenic environment of the oral cavity, can lead to disease recurrence or postoperative infections [36]. While previous studies have highlighted the inhibitory effects of nano-ZnO/HAw on aerobic and micro-aerobic bacteria [35], its impact on anaerobic bacteria remains unexplored. HA is frequently employed in oral bone defect treatments within anaerobic environments. *P.gingivalis*, an anaerobic bacterium, is a key pathogen responsible for periapical diseases, often culminating in periapical bone absorption [36, 47]. Therefore, we investigated the antibacterial properties of nano-ZnO/HAw against *P.gingivalis* in this study (Fig. 3A–C). Our findings revealed that neither HAs nor HAw exhibited inhibitory effects on *P.gingivalis* colony formation or biofilm formation. This suggests that HAs and HAw lacked antibacterial properties, and HAw, with its high aspect ratio, did not influence the viability or biofilm formation of *P.gingivalis*. Conversely, 5% nano-ZnO/HAw and 10% nano-ZnO/HAw significantly inhibited the colony formation and biofilm formation of *P.gingivalis*. This indicates that HAw modified with 5% and 10% nano-ZnO particles possessed favourable antibacterial properties. Consequently, nano-ZnO/HAw holds promise for applications in repairing periapical bone defects, even in the presence of *P. gingivalis*.

Previous studies have highlighted the antibacterial efficacy of high aspect ratio surface topographies, such as titanium dioxide nanopillars [42], black silicon with nanoneedles [48], poly(ethylene terephthalate) nanocone arrays [49] and nanostructures on cicada wings [50]. The physical interactions between the high aspect ratio surface topographies and the attaching bacteria generate shear forces during bacterial movement, resulting in the bacterial membrane to be deformed or punctured. The high aspect ratio surface topographies cause mechanical damage to bacteria, thus achieving antibacterial effects [42]. However, the mechanical damage from high aspect ratio surface topographies to cells is seldomly reported. In this study, we investigated the effect of HAw with high aspect ratio and sharp morphology on the microscopic characteristics of osteoblasts. Osteoblasts stretched well in control and HAs groups. Surprisingly, the punctured osteoblasts could be captured when cells were co-cultured with HAw (Fig. 4A and B). The findings conclusively demonstrated that HAw with high aspect ratio and sharp morphology caused mechanical damage to cells. Additionally, the integrity of the cell membrane was indirectly assessed using LDH release assay and trypan blue staining. There were no significant differences in LDH release and the number of blue-stained cells between the control group and the HAs group, suggesting that HAs did not disrupt osteoblast membrane integrity. However, a significant increase in LDH release was observed when cells were co-cultured with HAw suspension, corroborated by more blue-stained cells in the HAw group compared to the control or HAs groups (Fig. 4D and Supplementary S4). These results affirm that HAw disrupted osteoblast membrane integrity, leading to LDH release into the extracellular environment and trypan blue dye penetration into cells. These findings align with SEM observations, where HAw was seen to penetrate osteoblasts and cause mechanical damage to cells (Fig. 4A). Differing from the suspension of HAw which inhibited osteoblast proliferation (Fig. 5A) and differentiation (Fig. 5B and C), for the cells co-cultured with the extract of HAs or HAw, without the intervention of physical morphology, at each time point, osteoblast proliferation (Fig. 6A) and differentiation (Fig. 6B) showed no significant differences among the HAs, HAw or control groups over time. These results underscore that HAw with high aspect ratio and sharp physical morphology disrupted osteoblast membrane and caused mechanical damage to cells, thereby impeding osteoblast proliferation and differentiation. The punctured osteoblasts could be captured when cells were co-cultured with the suspension of 10% nano-ZnO/HAw (Fig. 4A and B). Whereas, according to the results of LDH release assay, the released LDH in 5% nano-ZnO/HAw group and 10% nano-ZnO/HAw group was lower than that in the HAw group (Fig. 4D). Moreover, compared with the HAw group, fewer blue stained cells were observed when cells were co-cultured with 5% nano-ZnO/HAw and 10% nano-ZnO/HAw (Supplementary Fig. S4). The surface modification with 5% and 10% nano-ZnO particles successfully prevented osteoblasts membrane from being disrupted by HAw, indicating that the mechanical damage from 5% nano-ZnO/HAw and 10% nano-ZnO/HAw to osteoblasts was lower than that from pure HAw. Meanwhile, the osteoblasts differentiation *in vitro* (Fig. 5D and E) and new bone formation *in vivo* (Fig. 8A–D) in the 5% nano-ZnO/HAw and 10% nano-ZnO/HAw groups were higher than those in the HAw group. These results indicate that surface modification with nano-ZnO particles reduced the mechanical damage from HAw to cells, thereby mitigating the negative impacts of HAw on osteoblast differentiation and new bone formation. Furthermore, SEM (Fig. 1A) and EDS (Supplementary Table S4) results revealed that the content of nano-ZnO particles on the surface of 10% nano-ZnO/HAw was higher than that of 5% nano-ZnO/HAw, suggesting that HAw was wrapped more completely by 10% nano-ZnO particles. Consequently, the mechanical damage from 10% nano-ZnO/HAw to osteoblasts might be lower than that from 5% nano-ZnO/HAw. Thus, 10% nano-ZnO/HAw exhibited higher biocompatibility than 5% nano-ZnO/HAw (Fig. 5A, D and E). These results affirm that surface modification with nano-ZnO particles is an effective strategy to enhance the biocompatibility of HAw.

**Figure 9.**
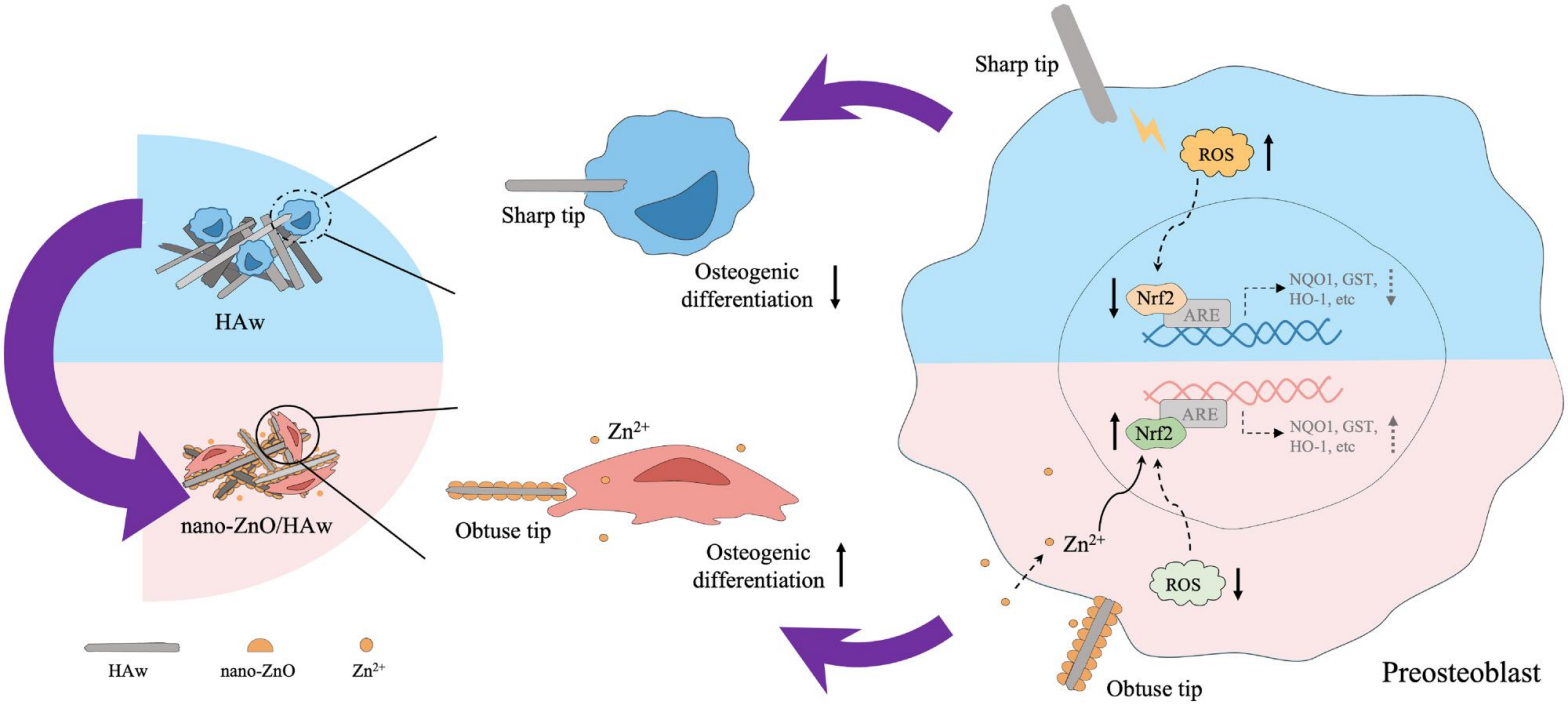
Scheme illustration of the mechanism of nano-ZnO particles surface modification improved the osteoinductivity of HAw.

ROS is a set of distinct molecular oxygen derivatives which produces during normal aerobic metabolism, and the balance between ROS production and elimination is essential for maintaining physiological functions [51]. Excessive ROS production can negatively affect osteoblast proliferation and differentiation [52]. Studies have shown that high aspect ratio surface topographies piercing into bacteria lead to a higher ROS level in bacteria [41, 42]. Osteoblasts could be punctured by HAw was confirmed in this study (Fig. 4A), and the intracellular ROS production of osteoblasts in the HAw group was higher than that of the HAs group (Fig. 7A and B). This suggests that the mechanical damage caused by HAw results in elevated intracellular ROS levels in osteoblasts, potentially negatively affecting osteoblast differentiation. Nrf2 plays a vital role in regulating the osteogenic differentiation of BMSCs and bone formation [44]. Studies have indicated that short-term ROS stimulation, induced by metal particles, transiently increases Nrf2 expression in BMSCs. However, prolonged peroxidation stimulation reduces Nrf2 expression and inhibits osteogenic differentiation of BMSCs. Activation of Nrf2 can alleviate the inhibitory effects of oxidative stress on antioxidant proteins and osteogenic marker genes expression in BMSCs [43]. In this study, the endonuclear Nrf2 expression of osteoblasts in HAw group was lower than that of HAs group (Fig. 7C). Taken together with the results that HAw promoted the intracellular ROS production of osteoblasts (Fig. 7A and B) and inhibited osteoblast differentiation (Fig. 5B and C), it suggests that HAw may decrease endonuclear Nrf2 expression by inducing ROS production in cells, thus negatively affecting osteoblast differentiation (Fig. 9). Conversely, the intracellular ROS production in osteoblasts co-cultured with the suspension of 10% nano-ZnO/HAw decreased compared to that in cells co-cultured with HAw (Fig. 7A and B). The endonuclear Nrf2 expression in the 10% nano-ZnO/HAw group was higher than that in the HAw group (Fig. 7C). Furthermore, the inducibility of nano-ZnO/HAw on osteoblast differentiation was higher than that of HAw (Fig. 5D and E). These results suggest that surface modification with nano-ZnO particles reduces the mechanical damage from HAw to cells, thereby inhibiting intracellular ROS production induced by HAw and mitigating its inhibitory effect on endonuclear Nrf2 expression in osteoblasts, ultimately promoting the osteoinductivity of HAw (Fig. 9).

Zn^2+^ plays an important role in promoting the osteoinductivity of biomaterials. Incorporating Zn has been identified as an effective strategy to enhance the biocompatibility of HA [53, 54]. For instance, the assembly of ZnO onto the surface of polyetheretherketone dramatically facilitates the cyto-activity of osteoblasts by releasing Zn^2+^ [30]. In this study, the extract of nano-ZnO/HAw effectively stimulated osteoblast proliferation (Fig. 6A) and ALP activity (Supplementary Fig. S6), suggesting that without the intervention of physical morphology and mechanical damage, the enhancement of osteoblast proliferation and ALP activity was related to the released Zn^2+^ from nano-ZnO particles. These findings are consistent with the results that the supplement of Zn^2+^ into the suspension of HAw increased osteoblasts differentiation (Fig. 7D and E). These results confirm that released Zn^2+^ from nano-ZnO particles enhances the osteoinductivity of HAw. Furthermore, the osteoinductivity of biomaterials exhibits Zn content dependency within a certain range [31]. With an increase in nano-ZnO content, the released Zn^2+^ from 10% nano-ZnO/HAw was higher than that from 5% nano-ZnO/HAw (Supplementary Fig. S2). Osteoblasts co-cultured with the suspension of 10% nano-ZnO/HAw exhibited higher proliferation (Fig. 5A), ALP activity (Fig. 5D) and osteogenic marker genes expression (Fig. 5E) compared to those co-cultured with 5% nano-ZnO/HAw. These findings demonstrate that the increased liberation of Zn^2+^ leads to a higher biocompatibility of 10% nano-ZnO/HAw than 5% nano-ZnO/HAw. Meanwhile, the cellular antioxidant activity is related to Zn. Under the stimulation of electrophilic substances, such as Zn^2+^, Nrf2 translocates to nucleus and binds to the promoter of antioxidant response element in nucleus, thereby promoting the expression of downstream antioxidant proteins of Nrf2 [32, 55]. Previous studies have shown that Zn ameliorates abnormal antioxidant capacity and attenuates arsenic overdose-induced brain damage by activating the Nrf2/Keap1 pathway [56]. Supplementation with Zn^2+^ promotes Nrf2 expression in preosteoclasts, reducing intracellular ROS production and inhibiting osteoclastogenesis [57]. In this study, the endonuclear Nrf2 expression in the HAw + Zn^2+^ group was higher than that in the HAw group (Fig. 7C), indicating that released Zn^2+^ from nano-ZnO particles effectively rescued the endonuclear Nrf2 expression in osteoblasts impaired by HAw. Additionally, compared to osteoblasts treated with HAw alone, intracellular ROS production decreased (Fig. 7A and B), and osteoblast differentiation significantly increased (Fig. 7D and E) upon supplementation of Zn^2+^ into the HAw suspension. These results suggest that the activation of Nrf2 by Zn^2+^ improves cellular antioxidant capacity, reducing ROS production induced by HAw and mitigating its negative impact on osteoblast differentiation. Notably, as depicted in Fig. 7D and E, ALP activity and osteogenic marker genes expression in osteoblasts co-treated with HAw and Zn^2+^ were intermediate between those of the HAw and 10% nano-ZnO/HAw groups, indicating that released Zn^2+^ partly counteracted the inhibitory effect of HAw on osteoblast differentiation. In summary, as illustrated in Fig. 9, nano-ZnO particle surface modification reduces mechanical damage from HAw to cells, combined with the promoting effect of the released Zn^2+^ on osteoblast differentiation, collectively enhances the osteoinductivity of HAw.

## Conclusion

Nano-ZnO/HAw was successfully developed, demonstrating remarkable improvements in the compressive strength and ductility of matrix materials. Furthermore, the surface modification with nano-ZnO particles mitigated the adverse effects of HAw on osteoblast differentiation by reducing mechanical damage to cells and releasing Zn^2+^. This released Zn^2+^ effectively restored impaired endonuclear Nrf2 expression caused by HAw, thereby enhancing osteoblast differentiation. Nano-ZnO/HAw exhibited favourable inducibility of osteoblast differentiation *in vitro* and promoted new bone formation *in vivo*. Overall, nano-ZnO/HAw showed promising osteoinductive properties while also displaying excellent reinforcing and toughening effects on matrix materials.

## Supplementary Material

rbae051_Supplementary_Data

## Data Availability

Data are available on request.
