# Supplementary material for: Nano-ZnO-modified hydroxyapatite whiskers with enhanced osteoinductivity for bone defect repair
Source: Regen Biomater. 2024 May 8;11:rbae051. doi: 10.1093/rb/rbae051 (PMC11162197; doi:10.1093/rb/rbae051)
Supplement: rbae051_Supplementary_Data [file rbae051_supplementary_data.docx]

**Supplementary materials**

**Nano-ZnO modified** **hydroxyapatite whiskers with enhanced**

**osteoinductivity for bone defect repair**

Penggong Wei^1, 2^, Ning Wang^3^, Qiyue Zhang^1, 2^, Wanfeng Wang^1, 2^, Hui Sun^1, 2^, Zengqian Liu^4^, Tingting Yan^5*^, Qiang Wang^1, 2*^, Lihong Qiu^1, 2 *^

^1^ School and Hospital of Stomatology, China Medical University, Shenyang 110002, China

^2^ Liaoning Provincial Key Laboratory of Oral Diseases, Shenyang 110002, China

^3^ The First Hospital of China Medical University, China Medical University, Shenyang 110001, China

^4^ Shi-Changxu Innovation Center for Advanced Materials, Institute of Metal Research, Chinese Academy of Sciences, Shenyang 110016, China

^5^ Faculty of Materials Science and Engineering, Kunming University of Science and Technology, Kunming 650093, China

*** Correspondence address:**

Email: [yan@kust.edu.cn](mailto:yan@kust.edu.cn) (T.Y.); mfqwang@cmu.edu.cn (Q.W.); [lhqiu@cmu.edu.cn](mailto:lhqiu@cmu.edu.cn) (L.Q.)

**The supporting information includes:**

• Supplementary Methods

• Supplementary Figures

• Supplementary Tables

**Supplementary Methods**

**Phase composition identification**

X-ray diffraction (XRD, Rigaku, Japan, D/max-2550v, Cu Kα radiation λ = 1.54056 Å) analysis was performed to identify the phase composition of nano-ZnO/HAw. The diffractometer was operated at 40 kV and 40.0 mA at a 2θ range of 20-60^◦^ with a step size of 0.026^◦^.

**ZnO content detection**

The content of zinc ions in nano-ZnO/HAw was determined using inductively coupled plasma atomic emission spectroscopy (ICP-AES; PerkinElmer, USA). The precise ZnO content of nano-ZnO/HAw was estimated accordingly.

**Zn^2+^ release behavior assay**

HAw, 1%nano-ZnO/HAw, 5%nano-ZnO/HAw, and 10%nano-ZnO/HAw were respectively suspended by α-MEM (40 μg/mL), and incubated at 37 ℃, 5% CO_2_. The supernatants were collected at 3, 5, 7, and 14 days. The concentration of Zn^2+^ was measured using inductively coupled plasma mass spectrometry (ICP-MS; Agilent, USA).

**Trypan blue staining assay**

Osteoblasts were seeded in 24-well plates at a density of 1 × 10^5^ cells per well. The cells were then co-cultured with suspensions (40 μg/mL) of HAs, HAw, 1%nano-ZnO/HAw, 5%nano-ZnO/HAw, and 10%nano-ZnO/HAw in α-MEM supplemented with 2.5% FBS, respectively. Cells cultured without HA samples served as the control. After 24 hours of incubation, the supernatant was removed, and trypan blue staining solution (Beyotime, China) was added to each well. Following a 10-minute incubation, the cells were observed under a microscope (Olympus, Japan).

**Cell apoptosis assay**

Osteoblasts were seeded in 6-well plates at a density of 4 × 10^5^ cells per well and co-cultured with suspensions (40 μg/mL) of HAs, HAw, 1%nano-ZnO/HAw, 5%nano-ZnO/HAw, and 10%nano-ZnO/HAw, respectively. After 2 days, cell apoptosis rates were measured using a flow cytometer (Becton Dickinson, USA) with an Annexin V-FITC apoptosis detection kit (Beyotime, China).

**ALP staining**

Osteoblasts were seeded in 24-well plates at a density of 5 × 10^4^ cells per well. The cells were then co-cultured with extracts of HAw, 1%nano-ZnO/HAw, 5%nano-ZnO/HAw, and 10%nano-ZnO/HAw in differentiation medium, respectively. After incubation for 3 and 7 days, the cells were fixed with 4% paraformaldehyde and stained using an ALP staining solution (Beyotime, China).

**Quantitative analysis of bone defect**

All rat femur specimens underwent Micro-CT scanning. A volume of interest (VOI) with a diameter of 3 mm and a height of 3 mm was selected as a cylinder from the cortical surface of the bone to the cancellous bone, corresponding to the dimension of the bone defect site. Components in the VOI consistent with the density of bone tissue were selected (threshold set at 65-150). The percent bone volume (BV/TV), trabecular number (Tb.N), trabecular thickness (Tb.Th), and trabecular separation (Tb.Sp) were quantitatively calculated using Micro-CT quantitative analysis software.

**
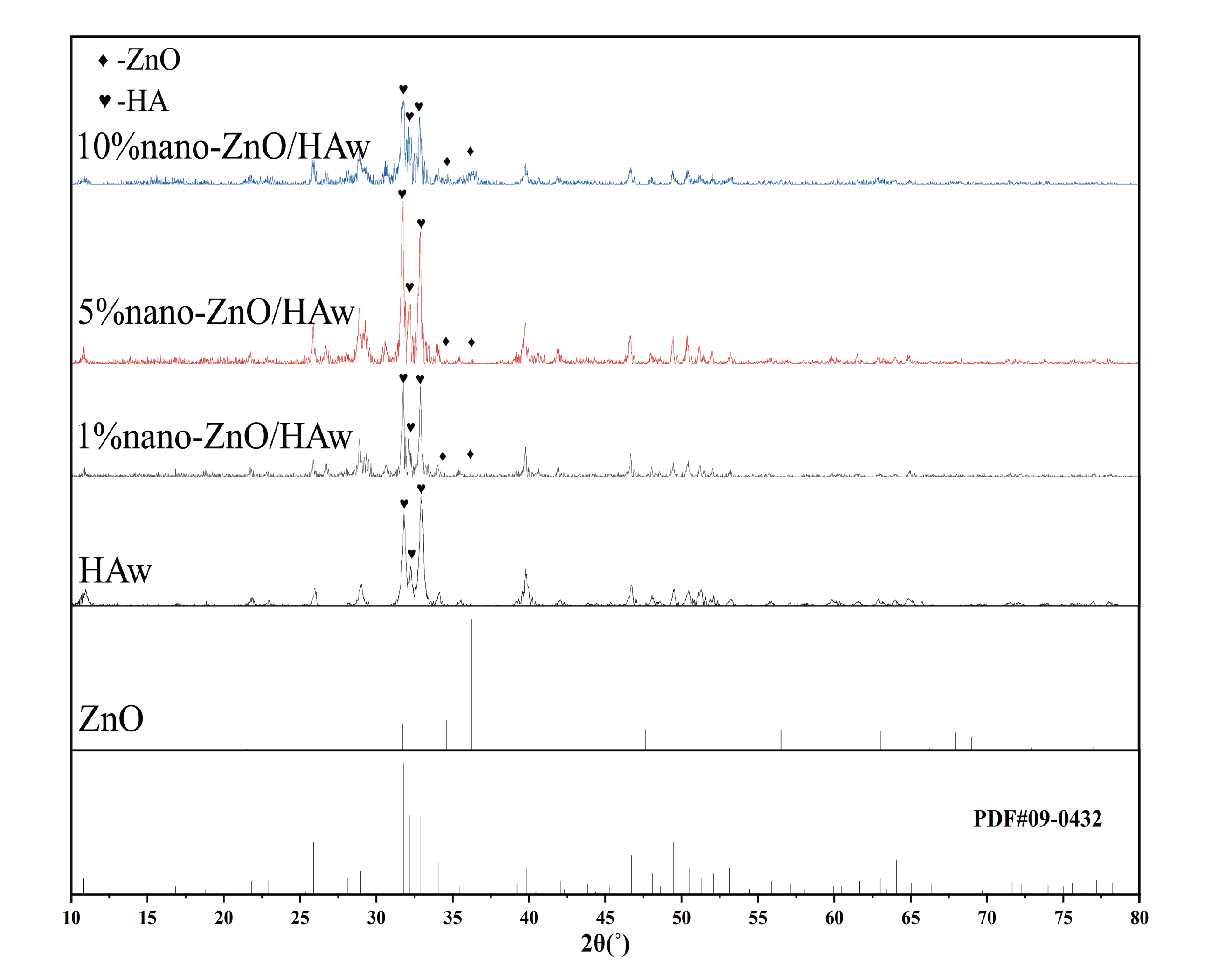
Supplementary Figures**

**Figure S1.** Phase composition of HAw, 1%nano-ZnO/HAw, 5%nano-ZnO/HAw, and 10%nano-ZnO/HAw.

**
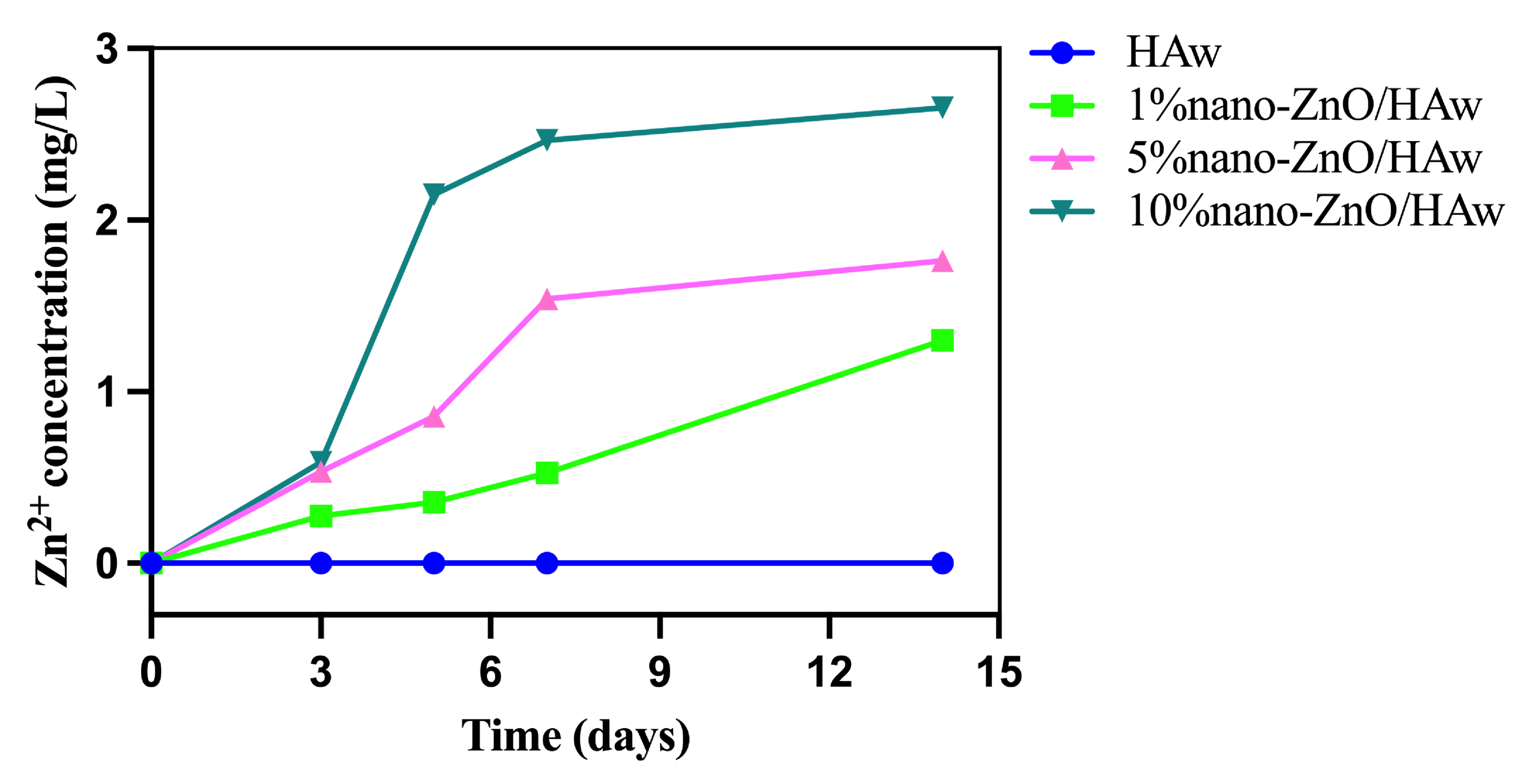
Figure S2.** Zn^2+^ release behavior of HAw, 1%nano-ZnO/HAw, 5%nano-ZnO/HAw, and 10%nano-ZnO/HAw at 3, 5, 7, and 14 days.

**
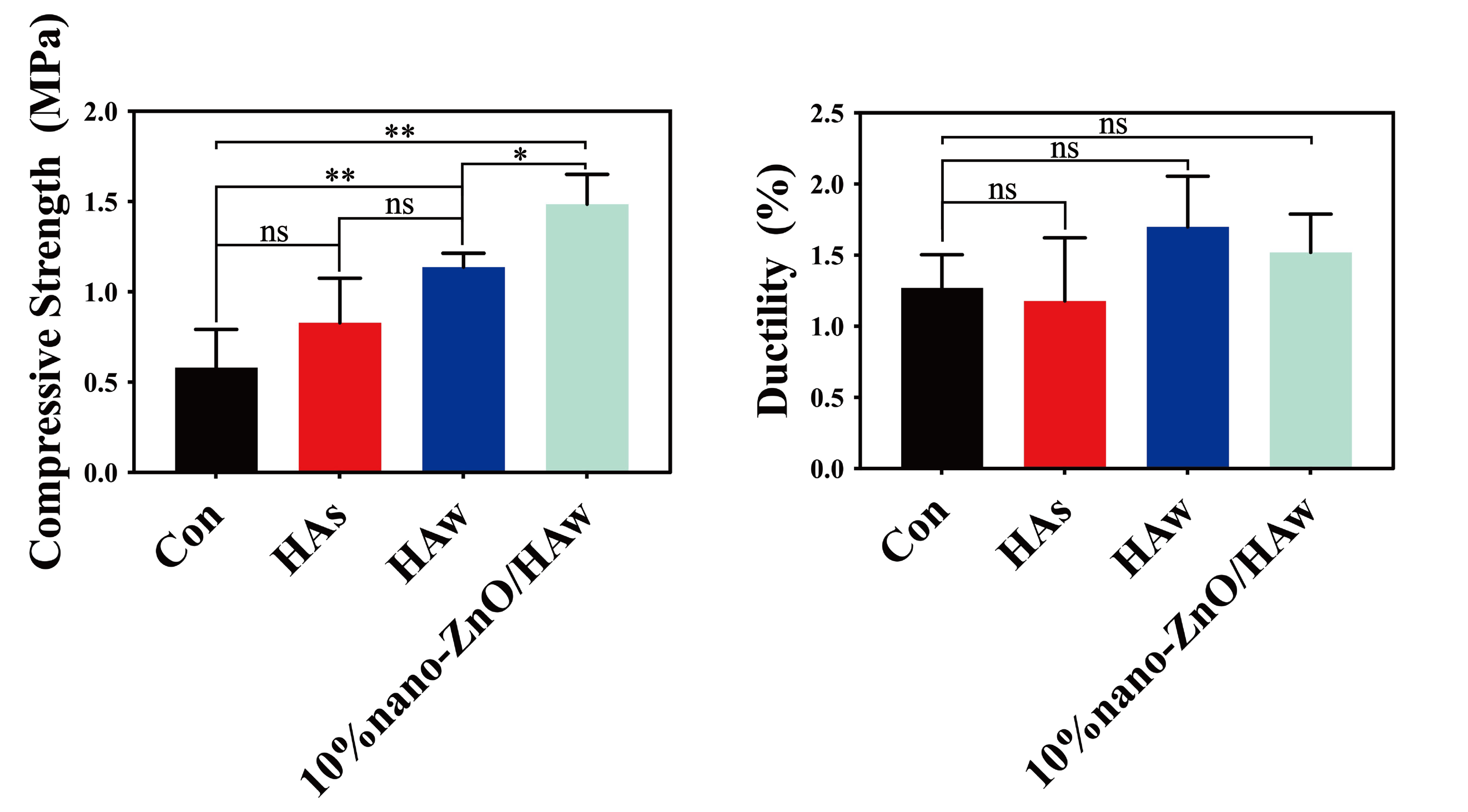
Figure S3.** Compressive strength and ductility of calcium sulfate incorporated with HAs, HAw, and 10%nano-ZnO/HAw. (* *P* < 0.05; ** *P* < 0.01; ns, no significance).

**
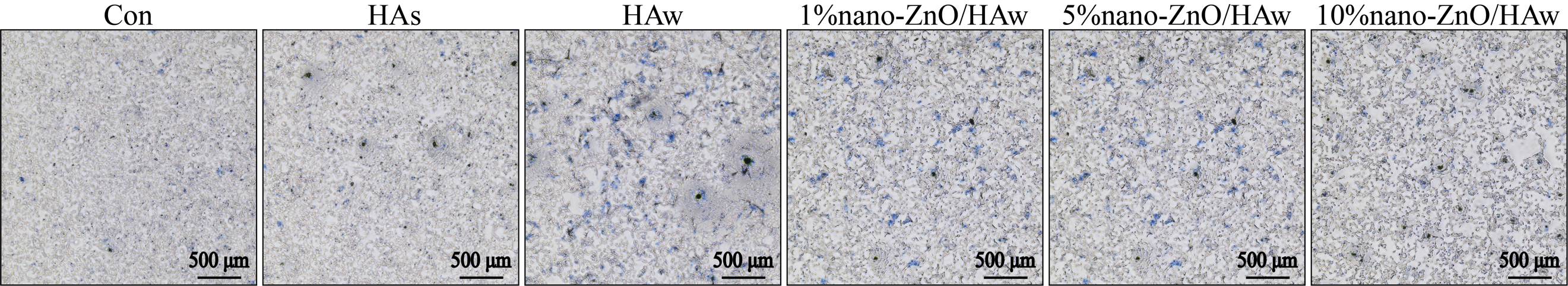
Figure S4.** Cells membrane disruption of osteoblasts co-cultured with the suspension of HAs, HAw, and nano-ZnO/HAw.

**
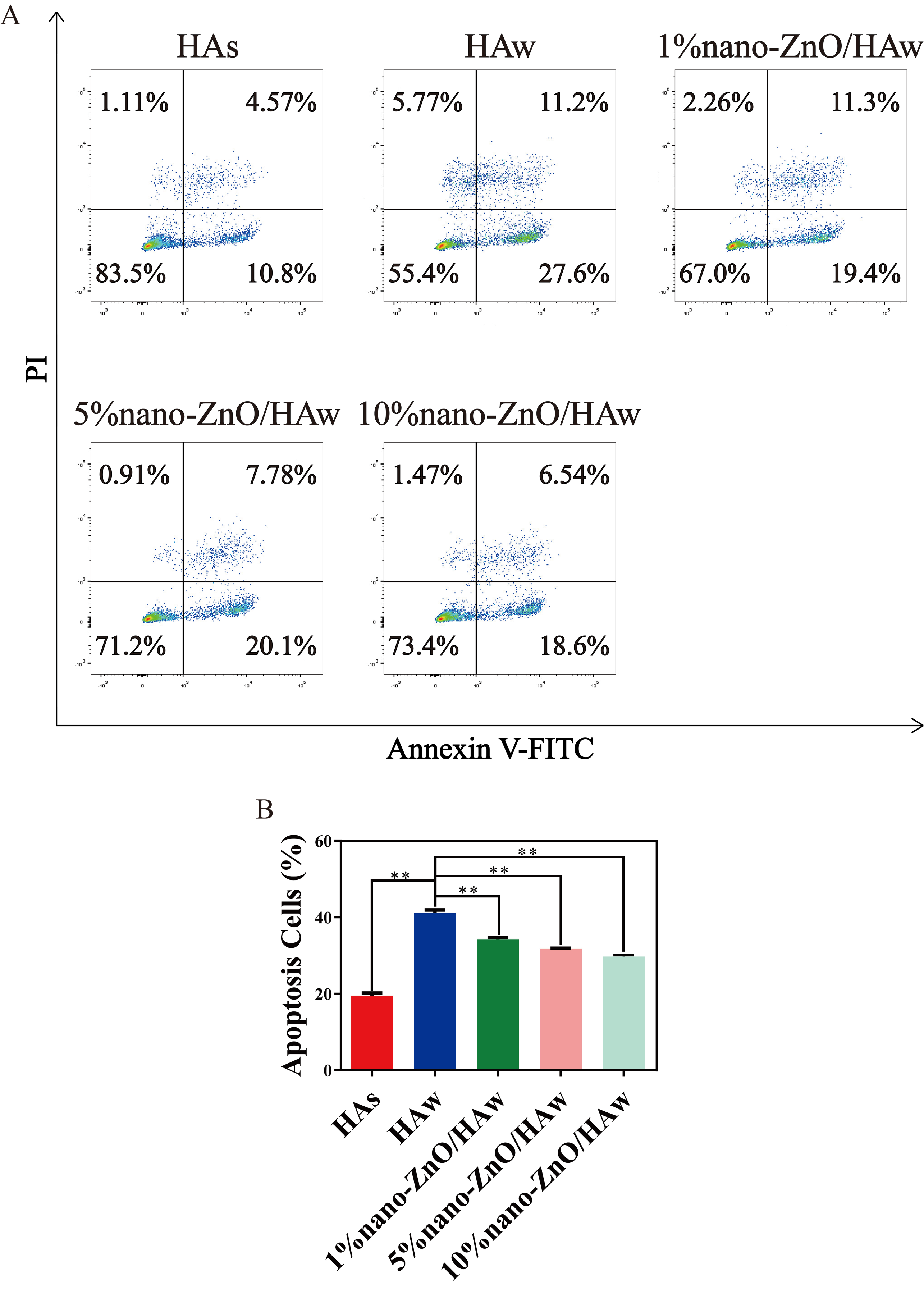
Figure S5.** (A, B) Cells apoptosis rate of osteoblasts co-cultured with the suspension of HAs, HAw, and nano-ZnO/HAw for 2 days. (* *P* < 0.05; ** *P* < 0.01; ns, no significance).

**
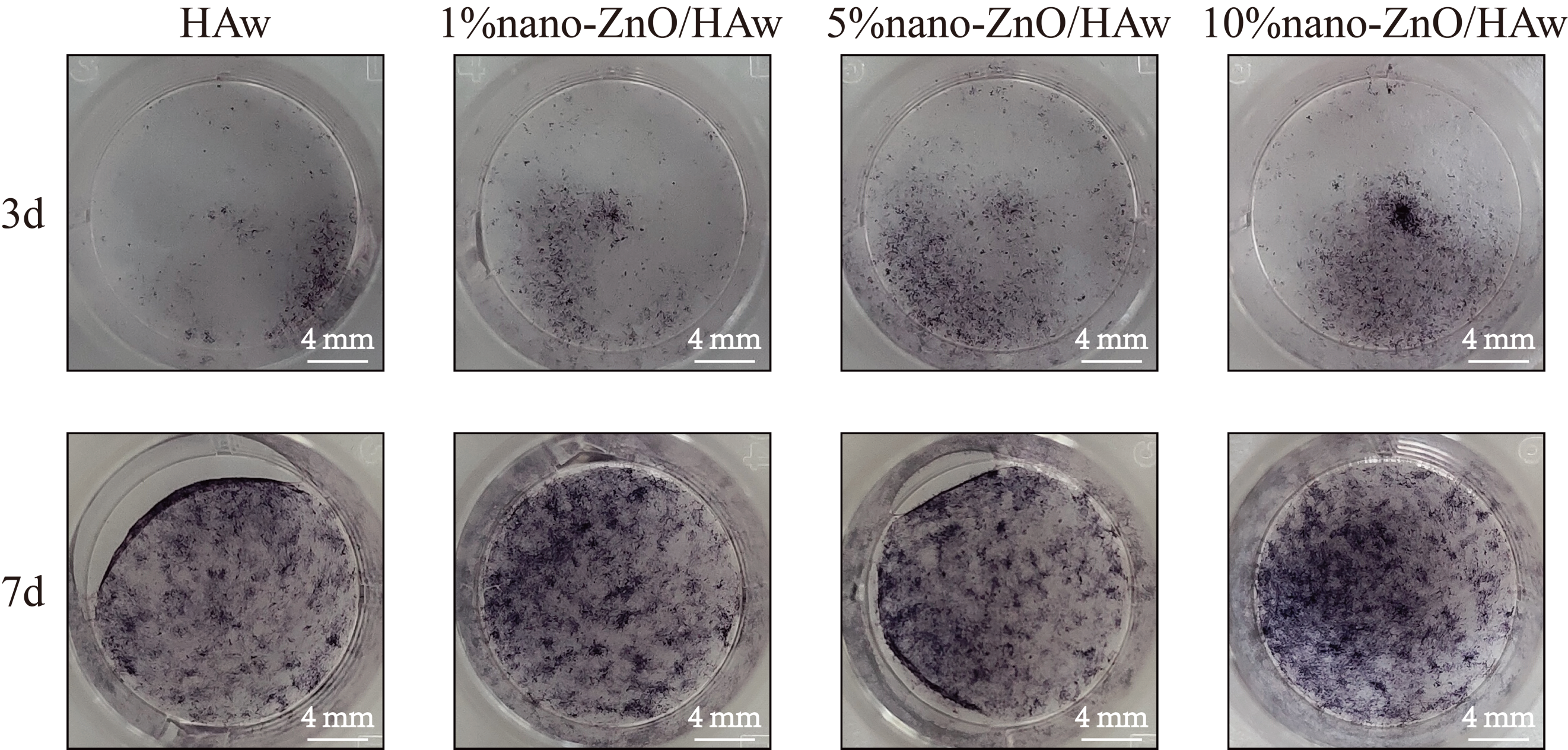
Figure S6.** ALP activity of osteoblasts co-cultured with the extract of HAw, 1%nano-ZnO/HAw, 5%nano-ZnO/HAw, and 10%nano-ZnO/HAw for 3 and 7 days.

**Figure S7.** Quantitative analysis of the components in cylindrical bone defect consistent with the density of bone tissue, including BV/TV, Tb.N, Tb.Th, and Tb.Sp. (* *P* < 0.05; ** *P* < 0.01; ns, no significance).


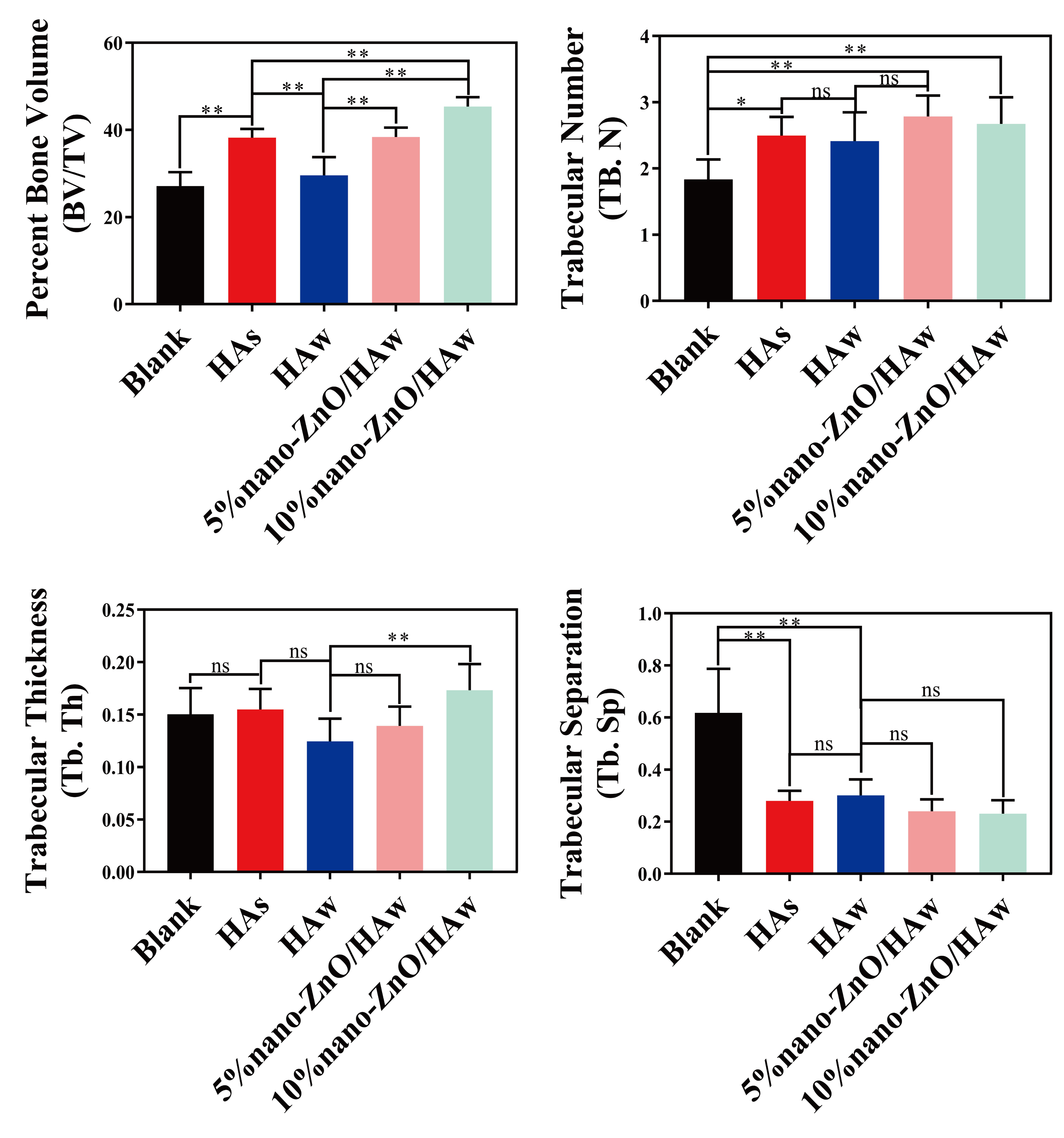


**Supplementary Tables**

**Table S1. Primers used in RT-PCR.**

| Forward (5’-3’) | | Reverse (5’-3’) |
| --- | --- | --- |
| GAPDH | TGTGTCCGTCGTGGATCTGA | TTGCTGTTGAAGTCGCAGGAG |
| ALP | AATCGGAACAACCTGACTGACC | TCCTTCCACCAGCAAGAAGAA |
| RUNX2 | CATTTGCACTGGGTCACACGTA | GAATCTGGCCATGTTTGTGCTC |
| OCN | CCGGGAGCAGTGTGAGCTTA | AGGCGGTCTTCAAGCCATACT |
| OSX | CTTCCCAATCCTATTTGCCGTTT | CGGCCAGGTTACTAACACCAATCT |
| COL I | CGATGGATTCCCGTTCGAGTA | GTGCTGTAGGTGAAGCGACT |

| **Table S2. Precise ZnO contents of nano-ZnO/HAw** | |
| --- | --- |
| Samples | ZnO content (wt%) |
| HAw | 0 |
| 1%nano-ZnO/HAw | 1.59 ± 0.01 |
| 5%nano-ZnO/HAw | 7.35 ± 0.03 |
| 10%nano-ZnO/HAw | 14.50 ± 0.09 |

| **Table S3. Sizes of HAw and nano-ZnO/HAw** | | | |
| --- | --- | --- | --- |
| Samples | Length (mm) | Width (mm) | Aspect ratio |
| HAw | 13.37 ± 2.38 | 1.68 ± 0.46 | 8.52 ± 2.68 |
| 1%nano-ZnO/HAw | 13.44 ± 3.92 | 1.58 ± 0.36 | 8.86 ± 2.91 |
| 5%nano-ZnO/HAw | 12.36 ± 2.77 | 1.60 ± 0.47 | 8.19 ± 2.81 |
| 10%nano-ZnO/HAw | 10.85 ± 1.89 | 1.61 ± 0.50 | 7.43 ± 2.92 |

| **Table S4. Surface elemental compositions of nano-ZnO/HAw** | | | | |
| --- | --- | --- | --- | --- |
| Samples | Ca (wt%) | P (wt%) | O (wt%) | Zn (wt%) |
| HAw | 31.45 | 14.09 | 54.46 | 0 |
| 1%nano-ZnO/HAw | 26.05 | 11.74 | 61.64 | 0.57 |
| 5%nano-ZnO/HAw | 30.96 | 13.71 | 50.21 | 5.12 |
| 10%nano-ZnO/HAw | 35.88 | 14.50 | 37.22 | 12.40 |
